# Human Immunodeficiency Virus-1 (HIV-1)-Mediated Apoptosis: New Therapeutic Targets

**DOI:** 10.3390/v6083181

**Published:** 2014-08-19

**Authors:** Zukile Mbita, Rodney Hull, Zodwa Dlamini

**Affiliations:** College of Agriculture and Environmental Sciences, University of South Africa, Florida Science Campus, C/o Christiaan de Wet and Pioneer Avenue P/Bag X6, Johannesburg 1710, South Africa; E-Mails: mbitaz@unisa.ac.za (Z.M.); hullr@unisa.ac.za (R.H.)

**Keywords:** apoptosis, HIV, HIV encoded proteins, antiviral, HIVAN, HAND

## Abstract

HIV has posed a significant challenge due to the ability of the virus to both impair and evade the host’s immune system. One of the most important mechanisms it has employed to do so is the modulation of the host’s native apoptotic pathways and mechanisms. Viral proteins alter normal apoptotic signaling resulting in increased viral load and the formation of viral reservoirs which ultimately increase infectivity. Both the host’s pro- and anti-apoptotic responses are regulated by the interactions of viral proteins with cell surface receptors or apoptotic pathway components. This dynamic has led to the development of therapies aimed at altering the ability of the virus to modulate apoptotic pathways. These therapies are aimed at preventing or inhibiting viral infection, or treating viral associated pathologies. These drugs target both the viral proteins and the apoptotic pathways of the host. This review will examine the cell types targeted by HIV, the surface receptors exploited by the virus and the mechanisms whereby HIV encoded proteins influence the apoptotic pathways. The viral manipulation of the hosts’ cell type to evade the immune system, establish viral reservoirs and enhance viral proliferation will be reviewed. The pathologies associated with the ability of HIV to alter apoptotic signaling and the drugs and therapies currently under development that target the ability of apoptotic signaling within HIV infection will also be discussed.

## 1. Introduction

Human immunodeficiency virus (HIV) is a retrovirus that causes Acquired Immunodeficiency Syndrome (AIDS) due to immune system compromise that leads to the body’s inability to fight off opportunistic infections. HIV infection results in the catastrophic loss of cells through apoptosis and chief among these are uninfected T cells, where the activation of programmed cell death pathways aid in the suppression of the immune system [1,2,3]. Apoptosis directed at the immune cells leaves the body helpless with no defense mechanisms. The devastating effects of HIV-1 infections are known, but how the HIV retrovirus manipulates the immune system in its favor remains elusive and unclear. Understanding the mechanisms that HIV-1 influences can lead to new therapeutic intervention and innovations.

The primary site of HIV infection is the lining of mucosal surfaces; a site that is abundantly inhabited by immature dendritic cells [4,5]. HIV infects all the CD4^+^ cells, including lymphocytes, monocytes and macrophages. These cells form the first line of defense, the innate immune system. In addition, HIV-1 persistently replicates in macrophages, compromising the integrity and function of the immune system, resulting in new HI viruses that further infect CD4 T cells, leading to severe immunosuppression [6]. However, while the percentage of lymphocytes declines, the percentage of macrophages does not. Since macrophages are resistant to the cytopathic effects of the virus, they survive for longer periods and contribute to further viral replication, serving as an important reservoir for HIV-1 persistence and replication [7,8]. CD8^+^ cytotoxic T lymphocytes kill the infected CD4^+^ cells, resulting in an immune-compromised body susceptible to opportunistic infections that can result in other pathologies. For new therapeutic interventions, the mechanisms that dictate the resistance or susceptibility of cells to HIV-induced apoptosis must be elucidated.

Proteins coded for by HIV have been implicated in the increase in cell death. These include gp120 [3,9] and Tat-proteins [10]. HIV induced apoptotic cell death was shown to occur in many different cell types [1,10]. These tissue specific increases in HIV induced apoptosis provide explanations for some of the diverse pathologies observed in HIV infected individuals. The apoptotic pathways induced by HIV are components of the intrinsic as well as the extrinsic apoptotic pathways and include the MAPK pathway [10], caspase 8 and 9 [11,12], destruction of host structural proteins [11], down regulation of BCL-2 [13], mitochondrial cytochrome c release [14], shortening of telomeres [15], HIV-1 long terminal repeats (LTR) and the transcription and activation of NF-κβ [16].

Currently, there is no cure for HIV infection. However, antiretroviral therapy against HIV has been very successful, but now research is focusing on achieving the ultimate goal, a cure. Consequently, understanding the host factors that control disease progression will be fundamental to the development of new therapeutic strategies [17]. In this review, the apoptotic pathways that result in the demise of the immune system and possible apoptotic-mediated interventions for therapeutic purposes will be covered. Finally, this paper will focus on the drugs currently under development.

## 2. Mediators of Apoptosis in HIV

**Figure 1:**
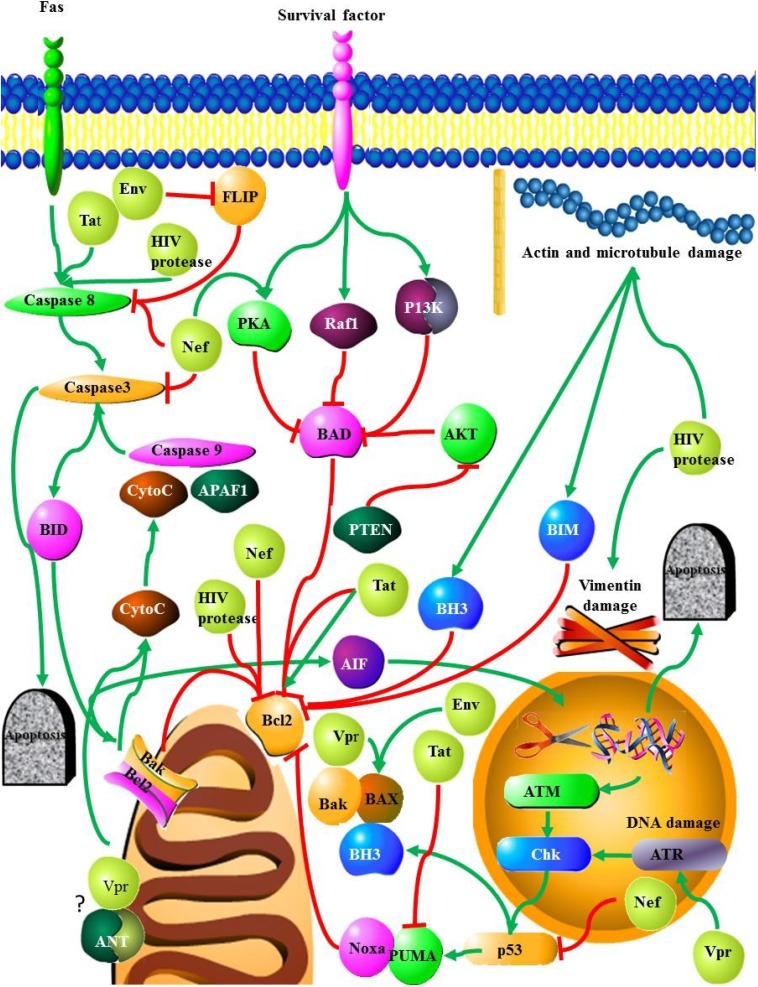
The involvement of HIV proteins in intrinsic apoptotic pathways: The viral proteins are marked in yellow. Bcl-2 is a central molecule in these mitochondrial-mediated cell death pathways. This protein is a target of Tat, Nef and HIV protease. Depending on the pathway activated the virus either shifts the balance of pro-apoptotic Bcl-2 family proteins to anti-apoptotic counterparts or vice versa. HIV proteins also affect the activation of caspases, and control their inhibitors as well, thereby, influencing the p53 signaling pathway.

**Figure 2:**
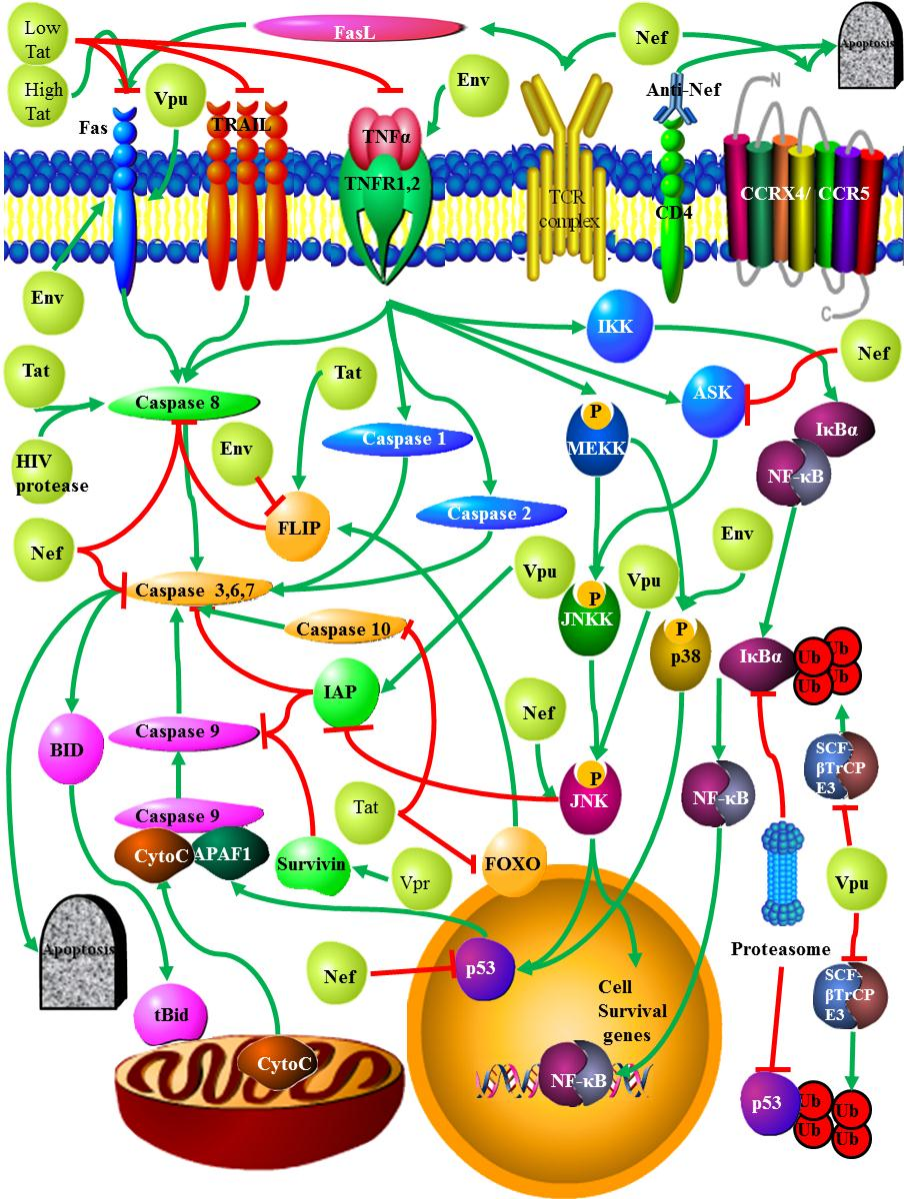
The influence of HIV proteins on the extrinsic apoptotic pathways: HIV proteins influence the expression of death receptors Fas or Tumor necrosis factor (TNF)–related apoptosis-inducing ligand (TRAIL)-R1/R1 (DR4/DR5). Up-regulation of these induces receptor—mediated cell death. HIV proteins also act on caspases, the p53 pathway and influence ubiquitin associated protein degradation.

Apoptosis occurs via two distinct pathways, an intrinsic (Figure 1) and an extrinsic pathway (Figure 2). The extrinsic (or external) pathway is initiated by the binding of ligands such as Fas ligand (FasL), TNF, and TRAIL/Apo-2 ligands to their death receptors FAS/CD95, TNFR1, DR4, and DR5 [18]. The pathway relies on the activation of caspases 8 and 10 which in turn activate the effector caspases 3 and 7. The intrinsic (or internal) pathway is initiated by the disruption of the mitochondrial membrane, resulting in the release of cytochrome c, regulated by the bcl-2 family of proteins [18]. The bcl-2 family contains pro- and anti-apoptotic proteins, with the pro-apoptotic proteins binding the anti-apoptotic proteins to initiate an apoptotic signal by promoting the dimerization of Bax and Bak [19]. These proteins function by creating pores within the mitochondrial; membrane. Once released cytochrome c is responsible for the assembly of a caspase-activating complex, which in turn activates caspase-9 [19]. While some of the HIV proteins such as Vpr can induce apoptosis in infected CD4 T cells via the mitochondrial pathway, (Figure 1), other HIV proteins (e.g., Nef) can induce apoptosis in CD4 T cells by the death receptor pathway (Figure 2).

### 2.1. Viral Entry and the Cell Surface Receptors

The primary and secondary cellular receptors CD4 and CCR5/CXCR4 are recognized by the viral envelope proteins. These envelope proteins then insert into the lipid membrane, and this is followed by the fusion of the viral and cellular membranes allowing entry of the viral particles. This fusion is facilitated by the fact that the viral envelope is formed when the viral particle, which is released through budding, consists of a portion of the plasma membrane of the host cell [20]. HIV-1 infection of CD4 T cells is favored by cell-to-cell contact, through the formation of the virological synapse [21]. It is important to note that both infectious and non-infectious HIV particles are able to induce selective apoptosis of CD4 T cells, suggesting that noninfectious HIV particles, which make up a large portion of plasma virus, contribute to the decline in CD4 counts in patients [22,23].

### 2.2. Death Receptors

The Tumor Necrosis Factor Receptor (TNFR) gene family contains the death receptor proteins. These molecules all contain a cysteine rich extracellular domain and a homologous cytoplasmic death domain. However, they do have diverse primary sequences allowing them to recognize specific ligands. The cytoplasmic death domains are responsible for the transmission of death signals through adapter proteins that contain death domains such as FADD, TRADD or DAXX [24].

#### 2.2.1. Fas/FasL

The Fas antigen is a member of the TNF-α family. Cross linking of this receptor led to apoptosis that is similar to that induced by TNF [25]. The Fas/Fas ligand (FasL) apoptotic pathway has been studied extensively, and it plays a major role in the induction of apoptosis in peripheral blood T cells [26]. Consequently, this pathway has been identified as a possible mechanism that contributes to apoptosis of CD4 T cells in AIDS [27]. Both soluble and membrane bound Fas and FasL are present at higher levels in HIV infected patients, and other studies showed that CD4 cross-linking and Fas ligation resulted in apoptosis of both CD4 and CD8 T cells [28,29]. The expression of the Tumor necrosis factor receptors Fas/FasL increases in human macrophages infected with HIV. HIV infection also up-regulates FasL in CD4 T cells after they are exposed to soluble Tat, gp120 or Nef proteins [30]. Treatment of HIV infected cells with Anti-Fas antibodies did not completely block apoptosis, which points to the involvement of multiple death pathways [22].

#### 2.2.2. TNF

Tumor Necrosis Factor α (TNFα) binds to either the 55 kDa TNFR1 or the 75 kDa TNFR2. Binding leads to the activation of transcription factors NF-κβ, AP-1, c-Jun N-terminal kinase, and p38. HIV proteins are known to bind to the Tumor Necrosis Factor Receptor (TNFR) which results in apoptosis in surrounding bystander T cells. Additionally, HIV-1 proteins target the TNFR pathway, altering gene expression and leading to increased HIV replication in infected cells [31]. The HIV-1 proteins Nef and Vpr both mimic TNFR signaling, resulting in the positive regulation of viral particle production (LTR activation) through the activation of NF-κβ and JNK pathways [31]. In the later stages of infection higher levels of TNF are produced leading to an increase in the death of uninfected bystander T cells, which gives rise to the subsequent immune suppression. TNFR stimulation leads to a decrease in the levels of the anti-apoptotic proteins Bcl-XL and activation of caspase 8 [32]. A model has been proposed explaining the interactions observed between TNF signaling and disease progression. In the initial stages of infection viral proteins activate the TNFR pathway to mimic the effects of this pathway. These include a G2 cell cycle arrest, the increase in changes in transcription leading to cell death, interlukin 1 secretion, cell proliferation and cell differentiation [33]. As the disease progresses the onset of AIDS is caused by viral proteins enhancing TNF signaling leading to an increase in apoptosis of bystander T cells. Pro-inflammatory cytokine production is also increased attracting more T cell and macrophages to the infected cell, further increasing the number of bystander cells that are induced to undergo apoptosis [31].

#### 2.2.3. TRAIL

Tumor necrosis factor (TNF)–related apoptosis-inducing ligand (TRAIL), a TNF super family member, induces the apoptosis of virus-infected and tumor cells. When exposed to HIV, uninfected CD4 T cells express TRAIL, DR5, and activated caspase-3 resulting in their eventual apoptosis [22]. Several other studies suggested a role for TRAIL in the apoptosis of CD4 T cells in HIV infection. For example, CD4 and CD8 T cells from HIV—infected patients were observed to be more susceptible to TRAIL-induced apoptosis *in vitro* than T cells from healthy donors [34]. TRAIL was also shown to induce selective apoptosis of uninfected CD4 T cells in HIV–infected human peripheral-blood leukocyte–non-obese diabetic–severe combined immunodeficient (hu-PBLNOD-SCID) mice [35]. TRAIL produced by monocytes exposed to the HIV Tat protein also resulted in the apoptosis of uninfected CD4 T cells [36].

The TRAIL protein is expressed on the cell membrane or secreted, and both the soluble and membrane-bound forms induce the apoptosis of cells expressing death receptors [37]. TRAIL has 2 death receptors capable of inducing apoptosis (DR4 and DR5), and 3 other receptors that engage ligands without initiating apoptosis [38,39]. The TRAIL gene is regulated by type 1 interferon (IFN)-α/β, which is mainly produced by plasmacytoid dendritic cells (pDCs) and has been shown to have a broad antiviral activity, including activity against HIV [40]. This apoptosis is partially prevented by anti-TRAIL antibodies, a situation similar to that observed for Fas/FasL and points to the involvement of multiple death mechanisms or receptors [22].

#### 2.2.4. Co-Receptors CCR5/CXCR4

In order for the virus to enter the host cells, the viral surface protein Env must first bind to the host receptor CD4 and consequently, to either the CCR5 or CXCR4 co-receptor, (Figure 1). CCR5 has three known natural ligands the presence of which reduces HIV infection by directly competing with Env for binding sites. These ligands: RANTES, MIP-1α and MIP-1β are produced by CD8^+^ T cells while CCR5 is expressed on the surface of macrophages, microglia and central and effector memory T cells [41]. CXCR4 is expressed on the cell surface lymphocytes [42], however, CXCR4 is more broadly expressed than CCR5 being found on the surface of most hematopoietic and parenchymal cells [41]. The physiological ligand for CXCR4 is the chemokine stromal cell-derived factor-1 (SDF-1) [42].

The T cell infecting strains preferentially induce apoptosis through interaction with CXCR4. Dual trophic strains have no preference for the co-receptor bound to induce apoptosis [43]. A change in HIV-1 bias for binding to CXCR4 over CCR5 precedes AIDS development and the decline in CD4 cell number. However, this co-receptor switch is not a requirement for disease progression [44], but CCR5 dependent apoptosis is an absolute requirement for the HIV-1 R5 trophic mediated killing of uninfected bystander cells [45]. Irrespective of the co-receptor used HIV is still able to induce TRAIL and DR5 expression and preferential apoptosis of CD4 T cells [22].

### 2.3. HIV Proteins and Apoptosis

HIV-1 encodes only 15 proteins [46] (Table 1) and thus must exploit multiple host cell functions for successful infection [47]. These include three structural proteins Gag, Pol and Env. These polyproteins are proteolysed to give rise to smaller individual proteins; Gag gives rise to four proteins MA (matrix), CA (capsid), NC (nucleocapsid) and p6. Pol gives rise to three proteins PR (protease), RT (reverse transcriptase) and IN (integrase). Finally, Env gives rise to two proteins SU (surface or gp120) and TM (trans-membrane or gp41). The remaining six proteins encoded by HIV include the two gene regulatory proteins Tat and Rev as well as the four accessory proteins Vif, Vpr, Nef and Vpu [48].

#### 2.3.1. HIV Protease

HIV protease is crucial for the life cycle of HIV, as this protease is responsible for cleaving the Gag/Pol polyprotein into functional subunits. Mutation or inhibition of this enzyme results in non-infectious viral particles [49]. Furthermore, HIV protease can also cleave proteins such as actin, laminin-B, desmon, vimentin; cleavage of vimentin results in changes in the nuclear morphology and chromatin organization [11,50,51]. The cleavage of these cytoskeletal proteins can result in apoptosis [11] **(**Figure 1**)**. Over expression of HIV protease in yeast and mammalian cells results in cell lysis with both cell types displaying a loss of plasma membrane integrity and changes in membrane permeability. In yeast cells the cell walls breakdown following HIV protease over expression [52].

The viral protease can also act on components of the apoptotic machinery, killing infected and uninfected lymphocytes through the action of several host molecules, by the extrinsic pathway through members of the tumor necrosis factor family (Figure 2), or via the mitochondrial apoptotic pathway [49] (Figure 1). Over expression of the viral protease in cultured cells results in increased apoptosis which can be visualized as increased DNA fragmentation. The increase in apoptosis observed here was due to the proteolytic degradation of Bcl-2 an anti-apoptotic protein by the HIV protease [53]. HIV protease is also able to cleave procaspase-8 into a unique form known as casp8p41 which is able to induce the mitochondrial-dependent pathway of apoptosis [54]. HIV protease can also lead to an increase in the levels of active NF-κβ through the proteolytic processing of the precursor molecule [55]. Additionally, NF-κβ can also be up-regulated through the actions of casp8p41 [56].

**Table 1:** Pro and Anti-apoptotic functions of HIV proteins.

	Pro/anti Apoptotic	Mechanism	Reference
HIV protease	Pro-apoptotic	Cytoskeletal damage	[51]
		Damage to Plasma membrane	[52]
		Proteolytic cleavage of Bcl-2	[53]
		Cleavage of procaspase 8	[54]
	Anti-apoptotic	Increased NF-κβ signaling	[55,56]
Tat	Pro-apoptotic	Up-regulate Caspase 3 and 8	[57]
		Up-regulation of FasL and RCAS	[58]
		Up-regulation of Bax	[58]
		Decrease in FOXO3a signaling- FLIP decrease	[59,60]
		Altered microtubule stability resulting in Bcl2 inhibition	[61,62]
		Increased ROS production	[63]
	Anti-apoptotic	Increased resistance of cells to TNF, Fas and TRAIL	[58]
		Decrease in Caspase 10	[64]
		Increase in FLIP transcription	[64]
		Increase in Bcl2 activity	[65]
		Decrease in FOXO3 leading to a decrease in Bim and Puma transcription	[66]
Nef	Pro-apoptotic	Up-regulation of FasL	[67]
		Increase in JNK signaling leading to increase in p53 transcription	[68]
		Decrease in Bcl-2 and Bcl-xL activity	[69]
		Binding to CXCR4	[70]
	Anti-apoptotic	Inhibition of caspase 3 and 8	[71]
		Inhibition of Ask1	[72]
		Phosphorylation of BAD	[73,74]
		Up-regulate MAPK and JNK	[75]
		Bind p53 and prevent p53 mediated apoptosis	[76]
		Down modulate the expression of molecules of the MHC class I	[77]
		PAK activation	[78]
		Inhibition of caspases 9 via increased nuclear export of TRNA via eEF1A and Exp-t	[79]
Vpr	Pro-apoptotic	ANT mitochondrial membrane permeability	[80]
		Bax activation	[81]
	Anti-apoptotic	Survivin	[82]
Vpu	Pro-apoptotic	Decrease in NF-κβ signaling	[83]
		Increase in p53 protein levels	[84]
		Increases the sensitivity of cells to Fas associated apoptosis	[85]
		Increase in JNK signaling	[86]
	Anti-apoptotic	Unknown role but cell type dependent decrease has been observed	[87]
Env	Pro-apoptotic	Up-regulation of Fas and Fas/L, and an increase in Fas mediated apoptosis	[88]
		Decrease in the transcription of the FLICE-like inhibitory protein (FLIP)	[89]
		Up-regulating Bax. Intrinsic apoptosis pathway	[9,89]
		Activation of the p38 but not AKT or ERK	[90]
		Induces membrane expression of TNF-α	[91]
		Hemifusion cell killing associated with caspase 3 and high ROS	[92,93,94]
		Syncitia formation leading fused cells to undergo apoptosis through the intrinsic pathway, involving the activation of Cdk1/cyclinB, Nk-κβ, mTOR, MAPK, p53 and PUMA	[95,96]
		Molecular mimicry of Fas	[97,98]
		Up-regulation of caspase 1	[99]
		gp-160 -CD4 complex blocks nuclear pores	[100]
		contagious apoptosis through caspase activation and alterations in mitochondrial trans-membrane potential	[101]
	Anti-apoptotic	High levels of CD4 expression lead to the retention of gp160-CD4 complexes within the Endoplasmic reticulum.	[102]

A list of HIV proteins and their pro and anti-apoptotic functions.

#### 2.3.2. Nef

The Nef accessory protein was originally thought to be a negative regulator of HIV replication, hence the name Negative factor (Nef). One of the primary functions of Nef and another accessory protein Vpu (discussed below) is the down regulation of CD4. Despite the fact that the CD4 receptor plays a critical role during viral entry, as continued expression of CD4 inhibits viral replication [103].

Nef is found as a soluble as well as a membrane bound protein on the surface of HIV infected cells, and facilitates its own secretion from infected cells by increasing the formation of exosomes [103]. Both versions are able to bind to CD4^+^ T lymphocytes and upon cross-linking by anti-Nef antibodies, the CD4^+^ T cells undergo apoptosis [104,105,106]. When Nef is added extra-cellularly it is also able to induce apoptosis in a variety of cell types by interacting with the chemokine receptor CXCR4 [70]. It has been reported that this death pathway relies on a protein kinase involved in apoptosis signaling that did not require Fas [69,107] or the activity of caspases [69]. Despite this, the over-expression of Nef in a T lymphocyte cell line led to an increase in the number of Fas/FasL molecules on the cell surface. This led to an increase in apoptosis which could be blocked using Fas/FasL blocking antibodies [67].

The induction of Fas ligand expression by Nef occurs following the direct binding of Nef to the T Cell Receptor Complex (TCR). This results in a TCR-Nef complex which is able to up-regulate Fas/L expression without the requirement for any specific antigen [77] (Figure 2). Transgenic *Drosophila* that over-express Nef revealed that Nef stimulated JNK dependent apoptosis and down-regulated the innate immune pathway mediated by Relish and NF-κβ [68]. Nef is also able to down-regulate the expression of anti-apoptotic proteins Bcl-2 and Bcl-X_L_ [69].

Nef plays an anti-apoptotic role in HIV infected cells giving time for viral particles to mature (Figure 1 and Figure 2). Nef was able to prevent apoptosis and up-regulate MAPK and JNK activities in the presence of TNF-α stimulation [75]. This characteristic of Nef to increase the resistance of cells to TNF-α induced apoptosis increases the pathogenesis of *Mycobacterium tuberculosis* infections, as cells infected with *M. tuberculosis* that would normally undergo apoptosis survive in an HIV positive background. Although evidence suggests that Nef and *M. tuberculosis* both activate TNF-α production individually, a combination of both leads to a larger decrease in TNF-α production. This implies that they activate TNF-α via different pathways [108]. Nef is also able to protect infected cells from Fas-mediated apoptosis by inhibiting the activities of caspase 3 and caspase 8 [71]. Nef associates with and inhibits apoptosis signal-regulating kinase 1 (ASK1), a serine/threonine kinase [72] and Nef is also able to bind p53 preventing p53 mediated apoptosis [76]. Serine kinase activity was found to be associated with Nef in T cell lines expressing hybrid CD8-Nef, as well as in lymphocytes infected with HIV [109]. This association and downstream effects of Nef signaling could be suppressed using serine/threonine kinase inhibitors [78]. The identity of this serine threonine kinase may be Nef-mediated p21-activated kinase (PAK) [110] or src like tyrosine kinases Hck and Lck, both of which are required for T cell activation [111]. It is thought that Nef associates with and activates PAK by forming a complex containing itself, PAK, phosphoinositide 3-kinase (PI3K), the small Rho-GTPases, Cdc42, Rac1, as well as a GEF providing factor. By activating PAK, Nef is also able to block the intrinsic apoptotic pathway by phosphorylating the pro-apoptotic Bad, leading to its dissociation from anti-apoptotic members of the Bcl2 family, decreasing apoptotic stimuli [73,74,78]. The Nef-Pak 2 interaction also plays a role in T cell development and motility as well as an increase in TCR signaling [78]. Nef is also able to decrease apoptosis by enhancing the nuclear transport of tRNA by translation elongation factor EF1A. The nuclear export of tRNA is performed via the Exportin pathway (Exp-1) through the direct interaction of aminoacylated tRNAs with eEF1A [112]. This elongation factor also plays a role in the regulation of the cytoskeleton by associating with actin and microtubules, and also plays a role in apoptosis. However, its role in apoptosis is conflicting, with some studies suggesting it increases the rate of apoptosis when expressed at higher levels, while other studies show eEF1A prevents cell death [112]. Nef was found to interact with eEF1A and increase its transport from the nucleus in an Exp-t dependent manner. Nef was able to rescue cells treated with the pro-apoptotic agent Brefeldin A (BFA) which acts by disrupting the cytoskeleton and increasing ER and Golgi apparatus stress. This is achieved by Nef increasing the nuclear export of NEF/eEF1A/tRNA complexes from the nucleus. The tRNA is then able to prevent mitochondrial cytochrome c release by binding to cytochrome c, inhibiting caspase-9 activation [79].

#### 2.3.3. Tat

The Trans-Activator of Transcription protein (Tat) is a small highly conserved protein that varies between 86 and 101 amino acids depending on the viral subtype [113]. Fas/FasL has also been shown to be up-regulated by Tat, which is secreted from infected cells, and can act on uninfected cells and enhance susceptibility to CD95-induced apoptosis [114]. The primary function of the HIV-1 Tat regulatory protein is the elongation of viral transcripts by facilitating the interaction of cellular factors with the long terminal repeat (LTR) viral promoter. Tat defective virus does not replicate efficiently in tissue culture. Cells infected with viruses lacking Tat show defects in elongation despite transcription beginning normally and results in few productive transcripts being generated [16].

Tat can modify the homeostasis of the immune system, by stimulating secretion of interleukin (IL)-1β, IL-4, IL-6, IL-8, IL-10, transforming growth factor (TGF)-β1, tumor necrosis factor (TNF)-α, and TNF-β [115,116]. In addition, Tat also induces the expression of the enzyme cyclooxygenase (COX)-2 and the synthesis of prostaglandin E2 (PGE2) [6]. Expression studies in both primary CD4 T cells and T cell lines highlighted the ability of Tat to induce apoptosis or to increase the sensitivity of the cells to pro-apoptotic signals [57]. Mutation studies also indicated that the ability of Tat to induce an apoptotic response was independent of its trans-activation and FasL functions, while expression studies revealed that Tat was able to induce apoptosis by up-regulating the expression of caspase 8 [57] and decreasing the phosphorylation of the forkhead promoter FOXO3a [59] (Figure 2). This will lead to a decrease in the expression of the anti-apoptotic caspase 8 inhibitor, FLIP [60]. Furthermore, the acetylation of Tat enhances its ability to bind and stimulate microtubule assembly which results in the formation of abnormally stable microtubules [61] leading to Bcl-2 dependent apoptosis [62]. Finally, Tat may be able to induce apoptosis through oxidative damage. Tat is known to increase the generation of reactive oxygen species, and Tat dependent apoptosis is inhibited by antioxidants such as N acetylcysteine (NAC). Tat also has the ability to decrease antioxidant defense systems, such as reducing catalase activity and reducing the ratio of GSH:GSSG [63].

The ability of Tat to inhibit the phosphorylation of Foxo3a may also play an anti-apoptotic role as this would inhibit the transcription of pro-apoptotic genes such as *Puma* and *Bim* [66]. Tat is also able to up-regulate the expression of the anti-apoptotic proteins Bcl-2 [65] and FLIP while down regulating the expression of caspase 10 [64] (Figure 2).

These conflicting roles of Tat in some pathways such as in the case of FOXO3a and FLIP may be due to the fact that the effect that Tat has on apoptosis seems to be reliant on the current levels of the protein. At low levels Tat decreases the sensitivity of cells to Fas, TNFα and TRAIL signaling [58]. At high levels Tat leads to an increase in the expression of FasL [117], caspase 8, Bax [58] and Receptor-binding Cancer Antigen expressed on SiSo cells (RCAS1). This receptor is similar to TRAIL, having a soluble and membrane bound form. Similarly, both forms can initiate cell cycle arrest and apoptosis in cells that express the receptor [118].

#### 2.3.4. Vpu

The Vpu protein is a 77–86 amino acid protein. Vpu enhances the release of mature virus from an infected cell [119], and is also able to down-regulate the expression of CD4 to allow for efficient viral replication and assembly. Phosphorylated Vpu is thought to bind to CD4 and to the SCF- βTrCP E3 ligase complex, resulting in polyubiquitination of CD4 leading to its degradation by the proteasome [120]. This allows Vpu to regulate the expression of Nf-κβ by interfering with the ability of the E3 ligase to transfer ubiquitin to phosphorylated Iκβ-α [83]. In a similar manner Vpu is able to interact with the E3 ligase complex and prevent the ubiquitination and degradation of p53. Consequently, this results in higher levels of active p53 and therefore, higher levels of apoptosis [84]. The ability of Vpu to interact with the SCF- βTrCP E3 ligase complex is conserved in *Drosophila* where Vpu binds to the homologous SLIMB/b-TrCP complex. As with mammals this results in an increase in apoptosis in the fly [86]. Vpu also increases the sensitivity of cells to Fas associated apoptosis [85], counteracts Tetherin, the host restriction factor active against HIV, through the ubiquitination of Tetherin in a manner similar to CD4 degradation [121].

Vpu has also been indicated in the E3 ligase independent up-regulation of apoptosis. Deletion of Vpu from HIV resulted in a decrease in the sensitivity of infected cells to Fas-induced death [85]. In the *Drosophila* model the expression of Vpu resulted in SCF- βTrCP dependent and SCF- βTrCP independent apoptosis. However, all forms of apoptosis relied on the activation of the JNK pathway by activating the upstream JNKKKs, DTAK1 and SLPR. This resulted in the expression of Reaper, resulting in the inactivation of Drosophila Inhibitor of Apoptosis1 [86].

Like other HIV proteins Vpu protein has also been shown to play a protective role against apoptosis. Two HIV isolates that demonstrated an increase in the levels of apoptosis they caused in uninfected peripheral blood mononuclear cells (PBMC), contained mutations in the *Vpu* gene. At the same time the *vpu* mutants induced a decreased apoptotic response in an infected CD4-T cell line. This demonstrated that the apoptotic response of cells to *vpu* mutants depended largely on the type of cell used [87].

#### 2.3.5. Vpr

HIV-1 Viral protein regulatory is a small 96 amino acid protein, the truncation of which results in a decrease in the replication rate of the virus. Vpr is responsible for the trans-activation of the HIV-1 LTR as well as for the import of pre-integration complexes. It is also responsible for the initiation of cell cycle arrest in the G2 phase [122], through ATR via the activation of γ-H2AX and BRCA1, and not through ATM or p53 [123]. Vpr also activates Wee the negative regulator of Cdk1 resulting in cell cycle arrest [124]. Vpr was also observed to associate directly with chromatin and may function by interacting with chromatin or with the replication machinery [122]. Alternatively Vpr could be interacting with the proteosome pathway as inhibition of this pathway inhibits the Vpr induced G2 cell cycle arrest [125].

Vpr is able to induce apoptosis through caspase 8 and 9 by causing sustained ERK activation [126] which is able to activate these caspases in a pathway that is FADD and Fas independent [127]. However, most Vpr mediated apoptosis occurs through mitochondrial injury, resulting in the release of cytochrome c and the activation of caspase 9. This occurs without the activity of caspase 8, the presence of Fas/L [128] or active p53 [129]. As inhibition of ATR in cells expressing Vpr resulted in the inhibition of cell cycle arrest and apoptosis, it was concluded that there was a relationship between the ability of Vpr to induce cell cycle arrest and its ability to induce apoptosis [130]. Some studies have concluded that the mitochondrial membrane permeabilisation that occurs during Vpr mediated apoptosis is due to the direct formation of large conductive pores due to the binding of Vpr to the Adenine Nuclear Transporter (ANT) [131]. It has been reported that Vpr acts directly on the mitochondria and its pro-apoptotic functions are not inhibited by the knockout of caspase activators Therefore, the ability of Vpr to induce apoptosis does not rely on caspase activation during the apoptotic response [132]. However, other studies used knockdown of ANT to show that the presence or absence of ANT did not affect Vpr mediated apoptosis. Rather Vpr induced apoptosis requires the activity of Bax. Vpr activates Bax through the upstream signaling pathway consisting of ATR and GADD45a, as the RNAi mediated silencing of either of these genes prevented Vpr induced apoptosis [133]. Vpr is also able to initiate mitochondrial permeabilisation by inducing the cleavage of BID to tBID [126].

Vpr can also function as an anti-apoptotic protein by activating the transcription of the inhibitor of apoptosis (IAP) *Survivin*. Survivin inhibits caspase activity and stops apoptotic signaling by Bax, TNF-*α* and Fas. The expression of Survivin is cell cycle dependent, with the highest expression occurring in the G2 phase. The establishment of a G2 cell cycle arrest by Vpr is the most likely mechanism behind the increase in Survivin expression [134]. It has also been reported that low level expression of Vpr protects cells from apoptotic stimuli. At these low levels there is no G2 cell cycle arrest. Therefore, like Vpu, Vpr plays different roles in apoptosis depending on its expression levels. With high levels leading to apoptosis and lower levels leading to an anti-apoptotic signal [135].

Vpr can reduce neuronal death by reducing the expression of pro-inflammatory cytokines such as IL-1β, IL-8 and TNF-α [136]. A large 1507 amino acid protein interacts with Vpr and was given the name Vpr Binding protein (VprBP). This protein is responsible for the regulation of p53 by binding to p53 promoter elements. This interaction between Vpr and VprBP allows Vpr to down-regulate p53 and therefore inhibit apoptosis [137].

#### 2.3.6. Env

**Figure 3:**
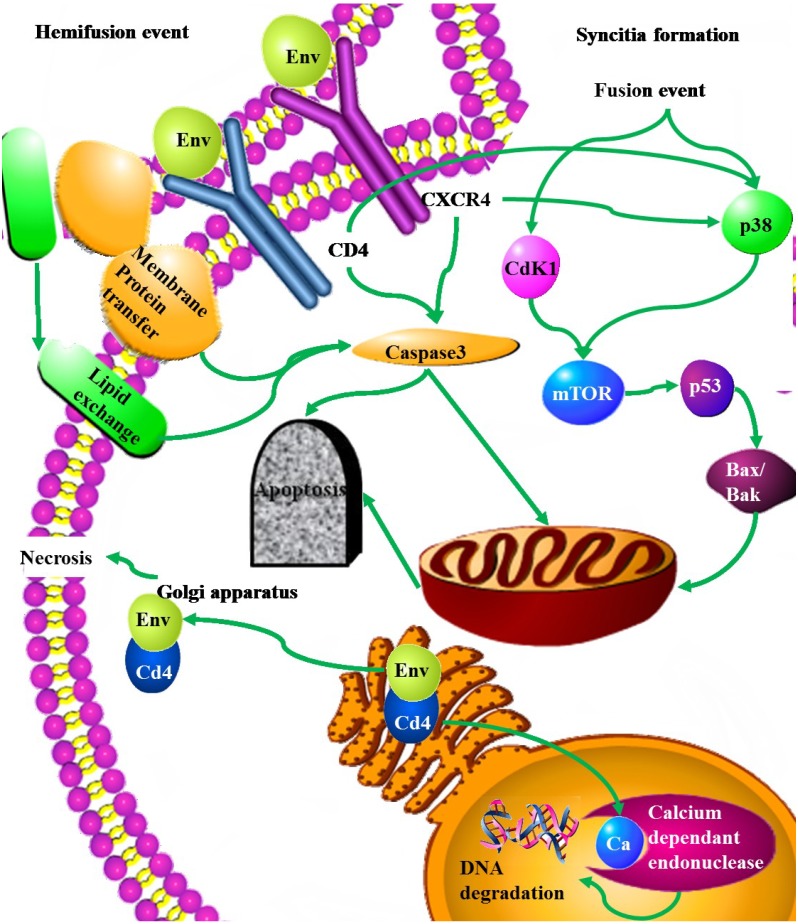
Mechanisms of apoptosis induction by the HIV-1 envelope protein at the cell surface. This can result from a hemifusion event through the transient interaction between Env- and CD4/CXCR4. This hemifusion event results in the exchange of membrane lipids and proteins. Alternately, Env-expressing cells can fuse with Env-negative cells resulting in the formation of a syncytium and death for the new fused cell. Here cell death relies on the p53 pathway via p38 and Cdk1 signaling.

The HIV Envelope protein is initially synthesized as a large precursor molecule known as gp160. This protein is then cleaved to give rise to the secreted gp120 and the trans-membrane gp40, Gp120 remains loosely associated with gp40 at the cell envelope [138]. Env contains a long signaling peptide that is located upstream of the region that forms gp120 following proteolytic cleavage. Replacing or removing this signal peptide resulted in decreased levels of apoptosis. This increase in apoptosis induced by the signaling peptide of Env was found to depend on the up-regulation of caspase 1 [99]. T cell apoptosis mediated by Env is due to impaired cytokine production, with Th1 cytokines such as IFN-g, IL-2 and IL-12 blocking apoptosis. The full length Env precursor gp-160 may associate with CD4 to form a complex that is capable of blocking the nuclear pores [100]. This leads to calcium ion build up within the nucleus and activates calcium dependent endonucleases, which cleave DNA resulting in apoptosis [138]. However, other studies indicate that this binding inhibits apoptosis as, high levels of CD4 expression lead to the retention of gp160-CD4 complexes within the Endoplasmic reticulum. At lower levels of CD4 expression the complex is transported to the Golgi apparatus where the gp160 is cleaved into gp120. The gp120-CD4 complex is then able to create pores in the membrane of the Golgi apparatus, migrate to the cell membrane and then to act in a similar way on the plasma membrane resulting in necrosis [102] (Figure 3).

The binding of gp120 to CD4 and CCR5/CXCR4 results in the transmission of death signals through these receptors leading to apoptosis. Alternatively, apoptosis may be achieved through membrane fusions. This may be either, a hemifusion event, or the formation of a syncytium [20]. Cross-linking of CD4 T cells with the envelope protein gp120 prior to T cell receptor (TCR) stimulation results in the up-regulation of Fas and Fas/L, activation the Fas/FasL (CD95/CD178) pathway and an increase in Fas mediated apoptosis [88]. Infected T cells co-express CD178 thus becoming potential killers of uninfected CD95-expressing T cells [67]. The binding of gp120 to CD4 separately from the TCR also results in a decrease in the transcription of the FLICE-like inhibitory protein (FLIP), which inhibits the proteolytic activation of caspase 8. In addition to this Gp120 and CD4 binding also activates intrinsic apoptotic pathways by up-regulating the pro-apoptotic Bax independently of Bcl-2. This then results in an increase in mitochondria-dependent apoptosis [89]. The activation of the p38 signaling pathway but not AKT or ERK is another reported outcome for the binding of gp120 to either CD4, or CXCR4. In the absence of AKT or ERK activation, p38 signaling leads to cell death, while the presence of AKT or ERK leads to cell survival [90]. Gp120 from HIV-1 and Fas, share a seven amino acid motif VEINCTR. This sequence is proximal to the immunogenic V3 loop of gp120 and antibodies found in the sera from HIV positive patients react with peptides containing this motif [97]. Due to molecular mimicry antibodies against gp120 bind Fas activating the Fas pathway and leading to apoptosis [98].

The association of Gp120 with CXCR4, results in a Fas independent death signal that leads to the depolarization of the mitochondrial cell membrane, cytochrome c release and the activation of caspase 9 [9]. Bystander T-cell killing can be mediated by macrophages since ligation of the chemokine receptor CXCR4 by gp120 or its ligand on macrophages induces membrane expression of TNF-α which triggers apoptosis on CD8^+^ T cells that express the receptor TNF-RII [91].

The association of Env with the CXCR4 receptor can result in cell-cell fusion between infected and uninfected cells [20] (Figure 3). Mutation studies revealed that the fusogenic activity mediated by gp41 is a requirement for the depletion of uninfected bystander T cells [139]. In order for this to occur, gp41 must mediate a close cell to cell contact. This then results in the death of the single uninfected cell through gp41 mediated transfer of lipids from the infected Env expressing cell to the uninfected cell [92]. This hemifusion cell killing can be inhibited by the over-expression of Bcl-2 [93] (Figure 3) and seems to be dependent on caspase 3 but not caspase 8.

Hemifusion apoptotic pathways also do not require p38 or p53 signaling and result in the generation of high levels of reactive oxygen species [94]. Membrane bound gp40 with associated gp120 binding to CXCR4 more commonly leads to the formation of short lived syncitia. These fusions between infected and uninfected cells lead to the cell undergoing apoptosis through the intrinsic pathway. It is unknown whether caspases are required for this apoptotic process with different studies reporting conflicting results [20,140]. The apoptotic cascade following cell fusion is initiated by the activation of Cdk1/cyclinB and NF-κβ. This is followed by an aborted entry into the prophase of mitosis where the nuclei of the cells fuse. At this stage p53 is phosphorylated by mTOR (Mammalian Target of Rapamycin) and cytoplasmic p38 mitogen-activated protein kinase (MAPK). Activated p53 then activates the transcription of Bax and Puma, activating mitochondrial apoptosis (95,96) (Figure 3). When apoptosis is initiated in cells expressing Env they can transfer this apoptotic signal to surrounding healthy cells. Different methods of inducing apoptosis in the donor cell still result in apoptosis in the target cell. In order for this contagious apoptosis to occur the donor cells have to be undergoing pre-apoptotic chromatin condensation. Target cells undergo apoptosis through caspase activation and alterations in mitochondrial trans-membrane potential [101].

## 3. HIV Related Apoptotic Pathways in Immune Related Cells

HIV-1 infects a variety of human cells with immune cells being the most commonly infected. These include CD4^+^ T cells, dendritic cells and macrophages. The first cells that HIV-1 encounters are dendritic cells, the potent antigen presenting cells that play a role in rapid infection of the T cells. Macrophages are known to be the major reservoir of persistent replication competent HIV-1 for extended periods, while Dendritic cells (DC) play a pivotal role in HIV-1 pathogenesis [141].

Dendritic cells (DC) are clustered in groups according to their location and are either blood DCs or are found in and around the skin and mucosal membranes. Blood DCs are subdivided into Myeloid DCs (myDCs) and plasmacytoid DCs (pcDCs) [142]. A normal immature DC cell serves to capture an antigen and migrate to the T cell areas of secondary lymphoid organs where it matures [143]. Blood DCs numbers are depleted upon infection which reduces IFN-alpha, resulting in a high viral load [142].

### 3.1. CD4^+^ T Cells

HIV infection is associated with a steady decline in the numbers of CD4^+^ T lymphocytes [144]. Under normal circumstances the pool of CD4^+^ cells is maintained through thymopoeisis and apoptosis [58]. Following viral infection CD4^+^ T cell numbers are depleted through increased apoptosis via multiple viral induced pathways. This increase in cell death is observed in both infected and uninfected cells. The levels of the bystander un-infected T cells that are lost due to apoptosis exceeds that of the infected T cells [58,144]. Additionally, both infectious and non-infectious HIV particles are able to induce selective apoptosis of CD4 T cells, suggesting that noninfectious HIV particles, which make up a large portion of plasma virus, contribute to the decline in CD4 counts in patients [22,23].

The primary and secondary cellular receptors CD4 and CCR5/CXCR4 are recognized by the viral envelope proteins. These envelope proteins then insert into the lipid membrane allowing the entry of the virus into the cell [20]. HIV-1 infection of CD4 T cells is favored by cell-to-cell contact, through the formation of the virological synapse [21]. Once the virus has infected the cell apoptosis can be initiated in a variety of means. These include the action of HIV protease which cleaves Bcl-2 and procaspae-8, with cytotoxic affects produced by other HIV proteins [145]. The cleavage of procaspase 8 performed by HIV-1 protease gives rise to a unique 41 kDa protein fragment known as Casp8p41. This fragment is observed to co-localise with infected and apoptotic cells. The levels of Casp8p41 correlates with CD4 cell count, with an increase in the level of Casp8p41 being associated with a decrease in the level of CD4 cells [12,146]. The generation of this HIV specific cleavage product seems to be necessary for the initiation of cell death in infected cells [12] as well as bystander T cells [147], and initiates cell death through the intrinsic apoptotic pathway involving the downstream activation of caspase 9 through the Bax/Bak facilitated release of cytochrome c [148].

The killing of uninfected bystander T cells occurs through the release of viral proteins gp120, Tat and Nef from infected. Gp120 triggers death in uninfected T cells by binding the receptors CD4, CXCR4 and CCR5 which leads to Fas up-regulation and a decrease in FLIP. This in turn leads to cell death through the intrinsic apoptotic pathway [145]. Tat enters the bystander cells and up-regulates TRAIL. TRAIL is induced by TLR on the surface of pcDCs which were shown to be able to kill CD4^+^ T cells. pcDCs presenting TRAIL were found in sites of high CD4^+^ T cell depletion [149]. HIV gp120 released from HIV infected DCs was implicated in T cell dysfunction [145,150]. Finally, Nef appears to kill uninfected T cells through the extrinsic and intrinsic pathways [145].

However, the main trigger for the death of these cells appears to be HIV-1 DNA products that arise due to the activity of the sterile alpha motif (SAM) domain and histidine—aspartate (HD) domain—containing protein 1 (SAMHD1). This enzyme acts to deplete dNTP stores and thus prevents complete DNA synthesis [151]. The presence of these viral particles is sensed by the interferon-γ—inducible protein 16 (IFI16) [152]. Unfortunately, although this enzyme protects these bystander cells from infection the resulting viral cDNA particles initiate apoptosis within these cells through the activation of caspases 3 and caspases 1. While caspase 3 initiates apoptosis through classical pathways, caspases 1 is involved in the innate immune response. Here the inflammasome initiates apoptosis through a process known as pyroptosis [153]. The levels of the detector IFI16 directly correlate with viral load as well as with the levels of cell death [152].

At the same time the failure of the virus to properly integrate into the host can also lead to apoptosis. The integration of viral cDNA requires DNA double strand break repair enzymes to repair single strand gaps between viral DNA and the target DNA as well as to circularise unintegrated viral cDNA. DNA damage is sensed by the molecular sensor ataxia-telangiectasia-mutated (ATM) which in turn activates DNA-dependent protein kinase (DNA-PK), The lack of either ATM or DNA-PK leads to errors in the integration of viral cDNA into host DNA [154,155]. The inhibition of the activity of DNA-PK leads to a decrease in the levels of HIV induced apoptosis. This implies that inhibition of DNA-PK by the virus would lead to a population of latently infected cells which do not produce new viral particles but still have the ability to do so [155]. This also implicates DNA-PK as a useful drug target [155].

### 3.2. The Contribution of HIV Proteins to Apoptosis in Macrophages

Macrophages are part of the cells of the innate immune system and along with CD4^+^ cells are the principal targets of HIV infection [74,145,156]. Viral entry seems to be modulated through CCR5, CD4 and CXCR4 surface proteins which also affects strain susceptibility [156]. In contrast to macrophages, peripheral blood monocytes are less frequently infected with HIV-1 *in vivo* [157]. Macrophages do not undergo cytotoxic events after infection and thus survive [158]. This is followed by the formation of vacuoles harboring viral particles [158]. These vacuoles known as virus containing compartments (VCCs) are present in uninfected macrophages but are more prominent following infection [145]. These surviving cells harboring viral particles act as reservoirs evading treatments such as HAART [74]. The ability of Macrophages to infect other cell types has a great effect on HIV pathogenesis. These cells include brain cells and T cells [159]. Direct transmission of virions to other cell types is through a virological synapse. This synapse is formed between an infected macrophage and uninfected cells expressing a receptor [159].

HIV encoded proteins play an important role in regulating apoptosis in macrophages. For instance, Nef elevated anti-apoptotic effects in the human macrophage precursor cell line. This enhances survival. Monocyte derived macrophages (MDMs) are involved in formation of HIV reservoirs affecting AIDS pathogenesis [74]. Tat can also enhance infectivity of HIV by recruiting monocytes, macrophages and dendritic cells. The expression of HIV co-receptors is also induced by the presence of Tat [145]. Vpu functions to degrade CD4 in the endoplasmic reticulum and counteracts host restriction, such as tetherin, to enable viral release. Degradation of CD4 follows its ubiquitination. A similar fate is dealt to tetherin. Vpu mutation hinders infection of macrophages [121]. Vpr is an absolute requirement for viral replication in macrophages through its ability to increase the activity of various transcription factors and to stimulate HIV replication [145].

## 4. HIV-Associated Pathologies: The Role of Apoptosis

Over 33 million people worldwide suffer from HIV/AIDS [160]. Highly active antiretroviral therapy (HAART) has extended the life expectancy of HIV-1 patients and has significantly reduced the viral burden [161]. Despite treatment serious HIV-1 related complications and pathologies have been shown to develop over time. These pathologies may include HIV-1 associated neurocognitive disorders (HAND), HIV-associated nephropathy (HIVAN), HIV-associated vascular complications and HIV-associated tumors such as Kaposi sarcoma [136] (Table 2). Although productive HIV-1 infection of primary neurons has not been demonstrated, HIV is able to affect neurons through indirect mechanisms. HIVAN is caused by infection of renal epithelial cells, where the kidney serves as a reservoir for HIV-1 [161,162]. It has been documented that 1% of HIV patients suffer from HIV-associated vascular complications [163]. Cardiovascular diseases progression is associated with the increased production of reactive oxygen species (ROS), such as superoxide. This leads to aberrant cell signaling, vascular smooth muscle cell hypertrophy and migration [164], endothelial dysfunction, and, potentially, apoptosis [165]. In people with HIV, cancer is a significant cause of mortality and morbidity, with 30%–40% of HIV patients developing a malignancy during their life time. Most of these HIV-related cancers (“AIDS-associated malignancies” or “opportunistic” cancer) are established as AIDS-defining and include Kaposi’s sarcoma (KS), non-Hodgkin’s lymphoma (NHL), and invasive cervical cancer (ICC) to name but a few examples.

**Table 2:** HIV related pathologies.

HIV-Associated Neurocognitive Disorders (HAND)		
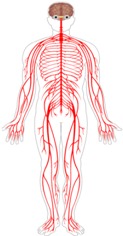	Prevalence 40%–50% of HIV-1 positive patients.	[166,167]
	Symptoms Impaired cognitive activity, memory, learning, attention, problem solving, decision making, confusion, forgetfulness, behavioral changes, and nerve pain.	[168]
	Histological patterns Macrophage infiltration, activated microglia, reduced synaptic/dendritic density and selective neuronal loss.	[169]
	Underlying causes Neuro-inflammation characterized by pro-inflammatory events Release of pro-inflammatory cytokines such as IL-1β, -6, TNF-α, and chemokines	[170]
Higher levels of TNF-α, IL-1β, IL-6, IL-8, monocyte chemo attractant protein-1, macrophage inflammatory protein-1 and CXCL10 are observed *in vivo* and *in vitro*.		[171,172]
Levels of these neuro-inflammatory factors are associated with higher viral load in cerebrospinal fluid (CSF).		[173]
Role of HIV proteins HIV-1 gene products are also known to modulate the levels of cytokines in macrophages.		
Tat stimulates cytokine/chemokine networks in monocytes and macrophages. Tat is also implicated in apoptosis using an excitotoxic mechanism to cause neurotoxicity. This excitotoxic mechanism involves the use of indirect and direct oxidative stress coupled with increased intra-cellular calcium and caspase3 activation.		[174] [166,175]
Further apoptosis is due to the mitochondrial release of cytochrome c and microtubule damage. This mechanism is similar to that induced by protease in lymphocytes with apoptosis as the end result		[14,166,176].
The surface of glial cells and neurons display CCR5 and CXCR4 which are targeted by gp120. This attachment, as in CD4 cells, causes apoptosis. An increase in gp120 concentration was shown to cause an increase in programmed cell death. Gp120 also inhibits the size and quantity of neurite growth and is known to activate caspases 3		[166] [167]
Vpr is the main viral protein responsible for neuropathology through pro-inflammatory cytokines.		[177]
The same proteins use similar mechanisms to cause apoptosis in both the nervous system and immune system. HIV-1 invades the central nervous system (CNS) during early infection via infiltrating monocytes and lymphocytes that are infected in the periphery		[178]
**HIV-Associated Nephropathy (HIVAN)**		
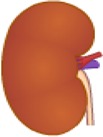	Prevalence Many patients with HIVAN ultimately progress to end stage renal disease (ESRD).90% of all ESRD cases attributed to HIVAN occur in African Americans	[179,180] [135]
	Symptoms	
	Inflammation is the major pathology.	[181,182]
Histological patterns Collapse of the glomerulus, cystic tubular dilatation and ultra-structurally actin cytoskeletal effacement. Histologic and molecular evidence of injury to glomerular podocytes. Normally terminally differentiated podocytes lose podocyte-specific proteins such as podocin and synaptopodin, and undergo proliferation and apoptosis		[183] [184,185] [186]
Underlying causes HIVAN is caused by infection of renal epithelial cells, where the kidney serves as a reservoir for HIV-1. Unlike neurons, HIV-1 can directly infect the renal tubular epithelium cells [RTEC] leading to HIVAN		[161,162],[187]
HIV-1 proteins interfere with signaling pathways that maintain cellular quiescence. The deregulated podocytes cannot re-differentiate into the quiescent state and are gradually depleted		[188,189]
Remaining podocytes undergo hypertrophy to cover a larger surface area resulting in denuded segments of the basement membrane that promote the development of sclerotic lesions.		[185]
Role of HIV proteins Vpr induces ERK, caspases-8 dependent apoptosis and hyperploidy in RTECs Nef protein activates ERK in podocytes. The ubiquitin-like protein FAT10 is up-regulated by HIV infection		[190,191] [190] [192]
**HIV-Associated Vascular Complications**		
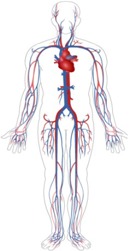	Prevalence 1% of HIV patients.	[163]
	Symptoms	
	Coronary heart disease, pulmonary hypertension (PH), and atherosclerosisIncrease the risk for noninfectious pulmonary conditions, including chronic obstructive pulmonary disease lung cancer pulmonary hypertension (PH).	[193,194,195] [196,197] [198]
	Underlying causes Increased production of reactive oxygen species (ROS), such as superoxide, leading to • aberrant cell signaling • vascular smooth muscle cell hypertrophy and migration • Endothelial dysfunction, and potentially apoptosis.	[164,165]
Role of HIV proteins Documented, but not yet fully understood. Suspected to play a role through promoting apoptosis, growth, and proliferation of a variety of cells *in vitro in vitro* by interacting with molecular partners in the infected host		[160,199,200]
**HIV-Associated Tumors, Kaposi Sarcoma (KS)**		
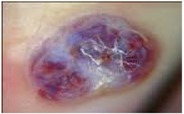	Prevalence For some time, Kaposi sarcoma was seen in 30%–40% of patients with AIDS, often as the presenting sign. The incidence of Kaposi sarcoma has fallen markedly in recent times, although its prevalence has not.	[201]
	Symptoms Raised red, purple, brown, or black blotches found on the skin, mouth gastrointestinal tract and respiratory tract.	[201]
Histological patterns Initially inflammatory dermatosis with signs of vasoformation. Later abnormal elongated spindle cells are present and are arranged in haphazard clusters. Dense vascularization with Hyaline globules		[202]
Underlying causes Up regulation of Bcl-2 expression associated with reduced endothelial cell apoptosis. Bcl-2 favors the angiogenic process which is switched oﬀ in healthy tissues Decreased expression of anti-apoptotic molecules occurs through the inhibition of endothelial cell adhesion onto the ECM or decreased expression of antigenic growth factors. The increase in Bcl-2 levels in late-stage KS lesions is accompanied with an increase in vascular cell apoptosis.		[203,204,205] [206,207,208,209] [210,211]
Role of HIV proteins The de-regulation of apoptosis by HIV proteins has been shown to play a significant role in tumor development. Tat eﬀects are cell-type dependent selectively promoting apoptosis in various cell systems. Tat increases Bcl-2 expression		[212,213,214]

This table lists the major HIV related pathologies that are linked to the ability of the virus to alter the patterns of apoptosis. It shows details of the prevalence of these pathologies, their symptoms, histology, underlying causes and the role played by the viral encoded protein products in contributing to the disorder.

## 5. Therapeutic Targets: Targeting Apoptotic Pathways

Various modulators of apoptosis have been identified over the years and strategies that target these regulators have been approved and applied in clinical trials. Both death receptors and classic apoptosis markers, such as Bcl-2 proteins, caspases and Bax, have been used as apoptotic therapeutic targets [215,216]. Caspases have been shown as early as 1999 to be effective in targeting HIV-infected cells [216,217]. Recently a caspase 3 (CASP3) has been engineered that is only activated by the HIV-1 protease. This protease was able to limit HIV replication in tissue culture by inducing apoptosis, implying that this mutant synthetic form of CASP3 could be used to treat resistant strains of HIV-1. Due to the mechanisms involved it was postulated that the possibilities of the HI virus developing resistance to CASP3 is unlikely [218].

The mTORC1 pathway was shown to be a therapeutic target for preventing the progression of HIVAN [219]. As previously stated HIV related renal failure is a result of tubular microcysts in cells leading to apoptosis, HIV-1 promotes renal tubular epithelial cell protein synthesis. The mTORC1 pathway plays an important role in mRNA translation and has been shown to be the cause of many renal diseases, and this pathway is activated in tubular cells upon HIV-1 infection [219]. Rehman *et al*. proceeded to inhibiting the pathway using rapamycin which resulted in diminished protein synthesis and thus no hypertrophy of the tubular cells [219]. Rapamycin is also commonly used to suppress the immune system as the mammalian target of rapamycin (mTOR) controls the activation and proliferation of T cells. Rapamycin can therefore reduce the number of T cells available for infection [220].

### 5.1. Therapies and Their Shortcomings

The ability of HIV to influence apoptotic pathways has allowed the virus to evade the immune system by blocking apoptosis of infected cells, by inducing apoptosis in other immune related cells or by evading the detection of infected cells. Two distinct types of therapies in relation to apoptosis have been approached [221]. Therapies based on the reactivation of latent virus aim to increase apoptosis in infected cells At the same time therapies aimed at blocking the apoptosis of immune related cells prevents the development of immunodeficiency [221,222].

Some potential reactivation therapies aimed at inducing apoptosis in infected cells include PI3K/Akt inhibitors that block HIV replication and favor apoptosis leading to the clearance of apoptotic cells [145]. Another strategy involves the use of IAP inhibitors in infected macrophages. Here HIV-1 infection of macrophages leads to an increased resistance of the macrophages to apoptosis through the expression of macrophage colony stimulating factor (M-CSF) which increases the expression of anti-apoptotic proteins while inhibiting the expression of death receptors. Conversely, the viral protein Vpr is also unable to induce apoptosis in macrophages. This can be reversed through the inhibition of IAP1 and IAP2 [145]. The use of Caspase 1 inhibitors such as VX-765, inhibits cell death due to the initiation of the IFI16 pyroptosis pathway and provides numerous advantages. Firstly they act on host pathways and would therefore avoid pathogen resistance. Secondly they are predicted to be clinically safe and finally, they are effective pyroptosis inhibitors. However, the value of pyroptosis inhibition in halting disease progression still has to be evaluated [152]. Another example of reactivation therapy involves the experimental use of Histone deacetylase inhibitors **(**HDACi) such as vorinostat, panobinostat and rhomedepsin. These inhibitors lead to increased DNA transcription and result in an increase in the levels of HIV RNA in resting memory CD4þ T cells. This will presumably increase the innate immune response leading to increased apoptosis in the infected cells. However, the predicted increase in apoptosis does not occur without the presence of an effective HIV-specific cytotoxic T-lymphocyte (CTL) response [222]. This is a common observation regarding reactivation therapy, where the inhibition of anti-apoptotic viral signals does not always result in the increase of apoptosis in infected cells and some evidence suggest it may actually increase viral load [221]. One method that could be used to overcome this is to sensitize the cells to apoptotic signals “priming” them for apoptosis [222].

Most conventional therapies, such as antiretroviral and protease inhibitors, generally decrease viral replication and the depletion of immune cells due to increased apoptosis, leading to a decrease in immunodeficiency. However, failures in these therapies due to the development of mutant viral strains, does not always lead to an increase in apoptosis [221]. This is observed in the use of enfuvirtide to inhibit gp41 fusion activity. Mutations develop that interfere with the ability of the drug to bind to gp41. These mutants also demonstrate a reduced ability to induce apoptosis in bystander cells due to reduced fusogenic activity [223]. Antiviral treatment also leads to HIV-1 protease mutations which inhibit the ability of the enzyme to cleave procaspase 8 into the pro-apoptotic Casp8p41. This results in a decrease in apoptosis [221]. Memory CD4 T cells also down-regulate procaspase 8 and demonstrate an intrinsic resistance to apoptosis [222]. Impaired pro-apoptotic ability as an evolutionary response of the virus to antiviral therapy may allow for increased viral replication in infected cells to compensate for decreased replication fitness [221].

### 5.2. Drugs Targeting HIV Proteins

The ability of HIV to cause pathologies as mentioned above led to many studies on elucidating the role that HIV proteins play in regulating apoptosis through molecular mechanisms, and signaling pathways. The practical application of all the information gathered is the development of a new generation of drugs that are more effective and have fewer side effects on patients. The different drugs that have been developed or are in the process of development include drugs targeting the effects of gp120, Tat-protein and the action of HIV protease (Table 3).

One strategy includes revisiting plant drug sources which are currently under-utilized with 36 plant families containing 46 plant species that have documented anti-HIV activity [224]. Many of these plant derived drugs were active against HIV protease, viral replication and syncitia formation by acting on gp120 and Tat-protein [224]. Since HIV protease, Tat-protein and gp120 are all implicated in apoptosis, these extracts potentially inhibit apoptosis.

**Table 3:** Drugs targeting apoptosis in HIV.

Drug	Target	Reference
Drugs targeting Tat		
Bovine Dialyzable Leukocyte Extract (bDLE)	Down regulation of Tat-protein lowers the expression of anti-apoptotic protein BCL-2 in infected cells	[81]
PI3K inhibitors and Akt inhibitors	Counter Tat-protein induced protection on infected cells	[82]
picolinic acid (PA) and fusaric acid (FA)	Target the conserved RING finger on Tat, inhibiting trans-activation	[225,226]
β-arrestin 2	Reduces apoptosis	[3]
Drugs targeting HIV protease		
Saquinavir, Ritonavir, Indinavir, Nelfinavir,Amprenavir, Lopinavir, Atazanavir, Fosamprenavir, Tipranavir, Darunavir	Inhibit HIV protease—inhibit viral maturation	[11,227]
Polyoxometalates	Act against HIV protease	[228]
Single-chain Fv (scFv)	An artificial derivative of mAb1696	
P27 peptide	Peptide derivative of the C- and N-terminal domains of HIV protease which inhibit dimerisation	[11]
mAb1696 antibody	Uncouples the protease dimer and induces inhibition	[229]
12-aminododecanoic acid (12-Ado)	Template for HIV protease dimerisation inhibition	[230]
C3-substituted cyclopentyltetrahydrofuranyl	Allosteric inhibitors Bind the flap region of HIV protease	[11,231]
GRL-02031	Another derivative of Cp-THF	[232]
Drugs targeting gp120		
Sifuvirtide	Fusion Inhibitor	[233]
Enfuvirtide	Fusion inhibitor	[41]
Maraviroc	CCR5 antagonist	[41]
4-phenyl-1-4-phenylbutyl piperidine (PPBP)	sigma-1 receptor agonist acting against gp120	[167]
Selective serotonin reuptake inhibitors (SSRIs)	Reduces cytokine receptor expression in the nervous system, reducing gp120 binding targets	[234]
Other drugs		
Double stranded RNA Activated Caspase Oligomerizer (DRACO),	Selects for viral infected cells only based on the length of RNA transcription helices. Increases apoptosis by caspase activation	[235]
NAPVSIPQ (NAP)	protects against mitochondrial release of cytochrome c.	[176]

This table lists some of the drugs currently under development that target the ability of HIV encoded proteins to manipulate the apoptotic machinery of the host cells.

#### 5.2.1. Drugs Targeting Tat

The immune system of HIV-1 patients was partially restored using bovine Dialyzable Leukocyte Extract (bDLE). This was even effective in individuals that were in the advanced stages of the disease. bDLE acts by preventing all stages of viral infection by excluding the HIV envelope on infected CD4 cells. This seems to be accomplished through the down regulation of Tat expression, resulting in lower levels of BCL-2 in infected cells [81]. PI3K inhibitors and Akt inhibitors are another class of drugs that target Tat by interfering with the ability of Tat to protect infected cells from apoptosis. The associated increase in apoptosis is not observed in uninfected cells [82]. Picolinic acid (PA) and Fusaric acid (FA) are two transition metals that have anti-HIV activity due to their ion chelating properties. This inhibits Tat trans-activation, most likely by targeting the conserved zinc finger of Tat [225,226], as chelation of the zinc finger results in a denatured, non-functional protein [226].

Investigations into the relationship between Tat protein and SIRT1, which plays a role in the activation of the p53 pathway leading to apoptosis in T-cells, [236,237,238,239,240,241,242] showed that Tat is able to inactivate SIRT1, thereby, activating the p53 pathway resulting in T-cell depletion [80,236]. This implies that SIRT1 could be used as a potential therapeutic target for inhibiting apoptosis [236].

#### 5.2.2. Drugs Targeting gp120

The inhibition or blocking of gp120 results in a decrease in syncitia formation or hemifusion events and therefore a decrease in gp120 dependent apoptosis. The entry of HIV into cells can be prevented by blocking gp120. This can be achieved through the over-expression of β-arrestin 2 which reduces apoptosis by down regulating *µOR* which in turn is up-regulated by gp120 [3]. Two drugs target gp120 in the nervous system 4-phenyl-1-4-phenylbutyl piperidine (PPBP) and Tianeptine. PPBP is a sigma-1 receptor agonist that partially inhibits gp120, significantly decreasing the apoptotic effects of the protein [167]. Tianeptine is a Selective serotonin reuptake inhibitor (SSRIs) that reduced cytokine receptor expression in the nervous system. With less receptors for gp120 to bind, caspase 3 activation is decreased resulting in reduced apoptosis [234]. The entry inhibitors Enfuvirtide (a gp41 derivative) and Maravirocis (a CCR5 antagonist) both have limitations. The effects of Enfuviritide in primary macrophages are not fully understood, while resistance to maraviroc has been observed. At the same time, other CCR5 inhibitors show serious side effects and a lack of clinical efficacy [145].

#### 5.2.3. Drugs Targeting HIV Protease

As previously stated the HIV protease initiates apoptosis by physically damaging the host cell’s structural proteins. HIV protease is also involved in viral maturation and assembly. However, so far attempts to accurately establish where this protease cleaves its substrates have been unsuccessful, complicating drug design. Inhibiting HIV protease has been effective in preventing viral maturation and damage to host proteins, resulting in a stall in apoptosis. This was shown using an HIV protease inhibitor saquinavir [243]. Current Protease inhibitors are competitive inhibitors using a structure based design. This increases the risk of resistance emergence, as they target the same active site. If resistance emerges, the danger is that this resistance may be to multiple protease inhibitors at once. One solution is the use of protease inhibitors that target other parts of the protease, namely: allosteric inhibitors. However, screening using structure-based design strategies has proved tedious due to high levels of toxicity at the cellular level. High throughput screening methods for allosteric inhibitors are required. The solution is the use of a cellular based system, which will allow for the screening of multiple attributes of a drug at the same time, including potency and toxicity. Potential compounds that are candidate allosteric inhibitors of HIV protease include; Polyoxometalates, C3-substituted cyclopentyltetrahydrofuranyl (Cp-THF) and dimerization inhibitors [11].

Polyoxometalates (POM) are oxide compounds from transition metals with a preference for tungsten, molybdenum and vanadium [244]. Early polyoxometalates were tested on HIV but had devastating side effects which resulted in the termination of drug testing [245]. However, less toxic POMs are being sought after. The mechanisms as to how they work are yet to be elucidated [246]. They were first tested for effect on reverse transcriptase which was negative [247]. Later they were shown to act against HIV protease [228], as the activity of POMs is easily interrupted by DMSO (Dimethyl Sulfoxide) which has previously been reported to stabilize HIV protease [246]. At least one POM has been reported to bind the hinge region of HIV protease. This kind of binding does not target the active site making POMs an allosteric inhibitor, which should prevent the development of resistance. Synthesis of POMs is also less laborious and more cost effective relative to organic inhibitors.

A specific class of POMs, Keggin and Dawson POMs are characterized by an organic side chain, and of the total of 28 POMs selected as potential drugs due to their known activity against HIV protease, six Dawson POMs and a single Keggin POM displayed total deactivation of HIV protease, showing that Dawson POMs are more efficient. A butyl side-chain trailed by a propionic acid side-chain enhanced activity among DMSO soluble POMs. NMR studies displayed very little change was caused by POM binding, thus a low-affinity binding is predicted [246].

The dimerization region of HIV protease is highly conserved making it a worthy target in HIV drug development [248]. The design of dimerization inhibitors was accelerated by the use of 12-aminododecanoic acid (12-Ado) as a template. 12-Ado permitted modification including directional changes and amino acid modification. These modifications displayed a cumulative effect with increasing potent modifications [230]. The β-sheet on the N-terminus of the protease monomer is most often targeted, although the C-terminus is also targeted [249,250]. Since partial dissociation of mature protease occurs, resulting in the disruption of the active site, dimerization inhibitors are classed as competitive inhibitors [251,252]. Inhibitors generally have low solubility due to being hydrophobic or containing a substantial hydrophobic constitution.

An antibody dimerization inhibitor known as mAb1696, uncouples the protease dimer and inhibits HIV protease activity [229]. Single-chain Fv (scFv) is an artificial derivative of mAb1696 retaining its potency at higher doses. The proposed mode of action involves detaching the N-terminal residues from the β-sheet resulting in a Hydrogen bonded loop. This allows for antibody binding preventing dimerization and thus disrupting the active site. scFv and mAb1696 both show activity againstHIV-1 and HIV-2 Proteases as well as a resistant variant. scFv prevents self-cleavage of HIV protease on the C-terminal but not on the N-terminus. The ability of mAb to bind to HIV protease is optimal on free N-termini, however, even small additions hinder its binding [229].

The C- and N-terminal domains of HIV protease themselves have inhibitory activity by preventing dimerization [250,253]. A synthetic peptide derivative of these termini fused with the Tat-protein cell permeable domain (CPD) is known as P27. P27 was active against protease and its resistant variant, R1, carrying eight resistance mutations. MTT assays on MT-2 cells showed protection of HIV-induced cell death in a dose dependent manner. Higher doses also led to decreased viral capsid formation, implying lower viral release. A combination of p27 and a potent active site inhibitor showed enhanced activity. Longer gag-pol peptides were observed signifying inhibition of viral maturation [253]. A-seco type triterpenoids are also protease inhibitors suspected to be dimerization inhibitors [254].

C3-substituted cyclopentyltetrahydrofuranyl (Cp-THF)-derived P2-ligands are inhibitors that bind the flap region of HIV protease. A derivative with a 3-(R)-hydroxy group displayed high inhibitory activity even against resistant proteases. Assays were conducted with various C-3 substituted polar group Cp-THFs to select a compound with maximum inhibitory and antiviral activity. By testing each of these modified Cp-THFs derivatives against a panel of proteases, including wild-type HIV1 and HIV2 with their resistant variants, an effective inhibitor can be identified [231,232,255,256,257]. Using this technique two compounds designated 3c and 3d were identified as being the most potent inhibitors of HIV protease. These two varied due to their stereo chemistry, with the more potent 3c displaying a unique water-mediated interaction with the backbone of HIV protease on the amide bond of Glycine 48 [231].

### 5.3. Other Drugs of Interest

Since peptides regulate most physiological processes, naturally synthesized peptides are an obvious source for new drugs. NAPVSIPQ (NAP) is a peptide derived from activity-dependent neuroprotective protein (ADNP). This peptide drug protects cells against the mitochondrial release of cytochrome c. Since it also stabilizes the Tau protein, NAP links the protection of microtubule network to the protection against apoptosis. NAP acts upstream of caspases 3 activation, preventing the release of cytochrome c [176].

Double stranded RNA Activated Caspase Oligomerizer (DRACO) is a broad spectrum antiviral drug. It selectively causes the apoptosis of virally infected cells. Healthy and infected cells are differentiated by the length of RNA transcription helices present in the cell. Viruses produce long dsRNA helices while the RNA helices of mammalian cells are much smaller. DRACO itself is a chimeric protein that binds viral dsRNA with one domain and binds pro-caspases with its other domain. If two or more DRACO molecules bind to the same dsRNA cells are then killed in a caspase dependent manner [235].

#### 5.3.1. Aptamers

Aptamers are small nucleic acids characterized by antiviral activity. The variation in aptamers is in what they bind. Targets include gp120, Tat-protein, HIV protease and Reverse transcriptase [258,259,260,261,262,263,264,265,266,267,268]. B40 is an RNA aptamer binding gp120, preventing viral entry. Real time surface plasmon resonance technology demonstrated that this was due to a disruption of CCR5 and gp120 interactions. B40 binds to the gp120 core, mutating gp120 core residues resulted in disruption of B40 binding. This binding also induces conformational changes affecting binding of other compounds [259,261].

The use of RNA aptamers provides for use of small interference RNA (siRNA). A siRNA aptamer against Tat-protein and a gp120 binding aptamer were tested *in vivo* [263], using RAG-humice which is a humanized mouse model which can sustain long term HIV infection [260]. The first molecule was an aptamer-siRNA chimera (Ch A-1). Ch A-1 was able to reduce viral load to undetectable quantities which lasted beyond treatment time. In contrast, the siRNA alone had no detectable effect on viral load. Both Ch-A-1 and the second apatamer, aptamer A-1, were able to significantly reduce cell death [263]. A preventative approach involves use of aptamer and siRNA chimeras to knockdown HIV target cells, *i.e.*, CD4 cells and macrophages within the host. This has proved effective in humanized mouse models [266].

The techniques of Nano-delivery as well as DNA aptamers have been formulated to address the instability of RNA aptamers, Targeted delivery would also improve the effectiveness of the drug [267,268]. Nanoparticle, pRNA, was used to deliver a siRNA chimera. This chimera targeted gp120 which is expressed on the surface of infected cells. This allows a gp120 specific aptamer to dock on gp120 expressing cells. The inclusion of Ab’ pRNA–siRNA chimera 2’-Fluoro backbone modifications of pyrimidines, stabilized the moieties from degradation and assisted in the activity of dicer in gene silencing [267]. The technique of systematic evolution of ligands by exponential enrichment (SELEX) allows the generation of siRNA carrying DNA aptamers. The DNA aptamer was generated through direct conversion of a CD4 specific RNA aptamer. These were used to perform siRNA against HIV protease. DNA aptamers with siRNA proved more stable and efficient in protease silencing than the RNA aptamer alone [268].

#### 5.3.2. Nanoformulation of ARVs

Targeted drug delivery promises to increase the effectiveness and safety of drugs. Multiple nano-delivery methods have been tested in the treatment of HIV. These include; polymeric nanoparticles [269], solid lipid nanoparticles (SLNs) [270], liposomes [271], nano-emulsions [272], dendrimers [273] and drug conjugates [274]. The size of Nano-delivery subjects is below one micron. Nano-delivery provides manageable toxicity patterns, adjustable drug release, low costs and high dosage tolerance. Additional advantages include protection from metabolism and a longer retention within the patient [275]. Active targeting allows for specific ligands on cell surfaces to be targeted, resulting in a specific delivery to a specific cell type. Additionally, the option to administer different drugs using one delivery system is available. This option eclipses error in administration and confers the ability to modulate individual drug release. The modes of delivery are specialized enough to allow Intracellular delivery [276], lymphatic system delivery [277] and central nervous system delivery [278]. With intracellular delivery lysosomal destruction can be bypassed allowing for nuclear or cytoplasmic delivery [276].

## 6. Conclusions

Despite advances in HIV treatments, there is still a high rate of infection and prevalence in sub-Saharan Africa, with the mortality rate due to HIV-infection being devastating throughout the world. In addition to this, the nature of HIV-infection may lead to other pathological condition such as tumourigenesis and HIV associated pathologies continue to affect patients despite the use of current treatments. Although it is currently the most effective treatment towards HIV-infection, discordant patients remain non-responsive to HAART. HAART also has side-effects such as lipodystrophy. The fact that the virus has developed the means to induce or inhibit apoptosis in ways that will benefit its survival has been a source of great interest and intensive study. By elucidating the mechanisms behind the ability of viral encoded proteins to alter apoptotic pathways, a long list of possible drug targets has been constructed. These vary from cell type specific signaling, to targeting interactions with cell surface receptors and components of the intrinsic and extrinsic apoptotic pathways. There are existing drugs that target the activity of HIV encoded proteins, such as HIV protease inhibitors. However, as mentioned above there is a large and varied list of different drugs using multiple approaches with a wide variety of targets that are currently under development. These drugs all share the common characteristic that they act to inhibit the ability of HIV to manipulate the apoptotic machinery of the host, thus, making it difficult for the virus to evade the immune system by decreasing the number of immune -competent cells by increased apoptosis. These drugs also decrease the ability of the virus to inhibit apoptosis in infected cells, allowing increased viral replication. By understanding and targeting the ability of the virus to manipulate the apoptotic machinery of the host these new therapies can aid to combat HIV/AIDS and improve the quality of life for HIV positive people including discordant patients.

## References

[1] Pantaleo G., Fauci A.S. (1995). Apoptosis in HIV infection. Nat. Med..

[2] Meyaard L., Otto S.A., Jonker R.R., Mijnster M.J., Keet R.P., Miedema F. (1992). Programmed death of T cells in HIV-1 infection. Science.

[3] Moorman J., Zhang Y., Liu B., LeSage G., Chen Y., Stuart C., Prayther D., Yin D. (2009). HIV-1 gp120 primes lymphocytes for opioid-induced, β-arrestin 2-dependent apoptosis. Biochim et Biophys Acta (BBA). Mol. Cell Res..

[4] Izmailova E., Bertley F.M.N., Huang Q., Makori N., Miller C.J., Young R.A., Aldovini A. (2003). HIV-1 Tat reprograms immature dendritic cells to express chemoattractants for activated T cells and macrophages. Nat. Med..

[5] Pontillo A., Silva L.T., Oshiro T.M., Finazzo C., Crovella S., Duarte A.J. (2012). HIV-1 induces NALP3-inflammasome expression and interleukin-1β secretion in dendritic cells from healthy individuals but not from HIV-positive patients. AIDS.

[6] Barreto-de-Souza V., Pacheco G.J., Silva A.R., Castro-Faria-Neto H.C., Bozza P.T., Saraiva E.M., Bou-Habib D.C. (2006). Increased Leishmania Replication in HIV-1 Infected Macrophages Is Mediated by Tat Protein through Cyclooxygenase-2 Expression and Prostaglandin E2 Synthesis. J. Infect. Dis..

[7] Brown H.J., Zack J.A. (2006). Animal models of HIV-1 latency and persistence. Curr. Opin. HIV AIDS.

[8] Zalar A., Figueroa M.I., Ruibal-Ares B., Bare P., Cahn P., de Bracco M.M., Belmonte L. (2010). Macrophage HIV-1 infection in duodenal tissue of patients on long term HAART. Antivir. Res..

[9] Roggero R., Robert-Hebmann V., Harrington S., Roland J., Vergne L., Jaleco S., Devaux C., Biard-Piechaczyk M. (2001). Binding of Human Immunodeficiency Virus Type 1 gp120 to CXCR4 Induces Mitochondrial Transmembrane Depolarization and Cytochromec-Mediated Apoptosis Independently of Fas Signaling. J. Virol..

[10] Gurwell J.A., Nath A., Sun Q., Zhang J., Martin K.M., Chen Y., Hauser K. (2001). Synergistic neurotoxicity of opioids and human immunodeficiency virus-1 Tat protein in striatal neurons *in vitro*. Neuroscience.

[11] Yang H., Nkeze J., Zhao R.Y. (2012). Effects of HIV-1 protease on cellular functions and their potential applications in antiretroviral therapy. Cell Biosci..

[12] Nie Z., Bren G.D., Rizza S.A., Badley A.D. (2008). HIV Protease Cleavage of Procaspase 8 is Necessary for Death of HIV-Infected Cells. Open Virol. J..

[13] Elmore S. (2007). Apoptosis: A Review of Programmed Cell Death. Toxicol. Pathol..

[14] Zou H., Li Y., Liu X., Wang X. (1999). An APAF-1 Cytochrome c Multimeric Complex Is a Functional Apoptosome That Activates Procaspase-9. J. Biol. Chem..

[15] Gaardbo J.C., Hartling H.J., Gerstoft J., Nielsen S.D. (2012). Incomplete Immune Recovery in HIV Infection: Mechanisms, Relevance for Clinical Care, and Possible Solutions. Clin. Dev. Immunol..

[16] Lin X., Irwin D., Kanazawa S., Huang L., Romeo J., Yen T.S.B., Peterlin B.M. (2003). Transcriptional Profiles of Latent Human Immunodeficiency Virus in Infected Individuals: Effects of Tat on the Host and Reservoir. J. Virol..

[17] Deeks S.G., Walker B.D. (2007). Human Immunodeficiency Virus Controllers: Mechanisms of Durable Virus Control in the Absence of Antiretroviral Therapy. Immunity.

[18] Lavrik I.N. (2010). Systems biology of apoptosis signaling networks. Curr. Opin. Biotechnol..

[19] Wood W.G., Igbavboa U., Muller W.E., Eckert G.P. (2013). Statins, Bcl-2, and apoptosis: Cell death or cell protection?. Mo. Neurobiol..

[20] Wan Z.-T., Chen X.-l. (2010). Mechanisms of HIV envelope-induced T lymphocyte apoptosis. Virol. Sinica..

[21] Piguet V., Steinman R.M. (2007). The interaction of HIV with dendritic cells: Outcomes and pathways. Trends Immunol..

[22] Herbeuval J.-P., Grivel J.-C., Boasso A., Hardy A.W., Chougnet C., Dolan M.J., Yagita H., Lifson J.D., Shearer G.M. (2005). CD4+ T-cell death induced by infectious and noninfectious HIV-1: Role of type 1 interferonâ-dependent, TRAIL/DR5-mediated apoptosis. Blood.

[23] Piatak M., Saag M.S., Yang L.C., Clark S.J., Kappes J.C., Luk K.C., Hahn B.H., Shaw G.M., Lifson J.D. (1993). Determination of plasma viral load in HIV-1 infection by quantitative competitive polymerase chain reaction. AIDS.

[24] Idriss H.T., Naismith J.H. (2000). TNFα and the TNF receptor superfamily: Structure-function relationship(s). Microsc. Res. Tech..

[25] Yonehara S., Ishii A., Yonehara M. (1989). A cell-killing monoclonal antibody (anti-Fas) to a cell surface antigen co-downregulated with the receptor of tumor necrosis factor. J. Exp. Med..

[26] Crisps N.I. (1994). Fatal interactions: Fas-induced apoptosis of mature T cells. Immunity.

[27] Dianzani U., Bensi T., Savarino A., Sametti S., Indelicato M., Mesturini R., Chiocchetti A. (2003). Role of FAS in HIV infection. Curr. HIV Res..

[28] Lelievre J.-D., Petit F., Arnoult D., Ameisen J.-C., Estaquier J. (2005). Interleukin 7 Increases Human Immunodeficiency Virus Type 1 LAI-Mediated Fas-Induced T-Cell Death. J. Virol..

[29] Oyaizu N., Adachi Y., Hashimoto F., McCloskey T.W., Hosaka N., Kayagaki N., Yagita H., Pahwa S. (1997). Monocytes express Fas ligand upon CD4 cross-linking and induce CD4+ T cells apoptosis: A possible mechanism of bystander cell death in HIV infection. J. Immunol..

[30] Poonia B., Pauza C.D., Salvato M.S. (2009). Role of the Fas/FasL pathway in HIV or SIV disease. Retrovirology.

[31] Herbein G., Khan K.A. (2008). Is HIV infection a TNF receptor signalling-driven disease?. Trends Immunol..

[32] De Oliveira Pinto L.M., Garcia S., Lecoeur H., Rapp C., Gougeon M.-L. (2002). Increased sensitivity of T lymphocytes to tumor necrosis factor receptor 1 (TNFR1) a and TNFR2-mediated apoptosis in HIV infection: Relation to expression of Bcl-2 and active caspase-8 and caspase-3. Blood.

[33] Friedmann E., Hauben E., Maylandt K., Schleeger S., Vreugde S., Lichtenthaler S.F., Kuhn P.H., Stauffer D., Rovelli G., Martoglio B. (2006). SPPL2a and SPPL2b promote intramembrane proteolysis of TNFα in activated dendritic cells to trigger IL-12 production. Nat. Cell. Biol..

[34] Lum J.J., Pilon A.A., Sanchez-Dardon J., Phenix B.N., Kim J.E., Mihowich J., Jamison K., Hawley-Foss N., Lynch D. H., Badley A.D. (2001). Induction of Cell Death in Human Immunodeficiency Virus-Infected Macrophages and Resting Memory CD4 T Cells by TRAIL/Apo2L. J. Virol..

[35] Miura Y., Misawa N., Maeda N., Inagaki Y., Tanaka Y., Ito M., Kayagaki N., Yamamoto N., Yagita H., Mizusawa H., Koyanagi Y. (2001). Critical Contribution of Tumor Necrosis Factor α Related Apoptosis-Inducing Ligand (Trail) to Apoptosis of Human Cd4+T Cells in HIV-1 Infected Hu-Pbl-Nod-Scid Mice. J. Exp. Med..

[36] Yang Y., Tikhonov I., Ruckwardt T.J., Djavani M., Zapata J.C., Pauza C.D., Salvato M.S. (2003). Monocytes Treated with Human Immunodeficiency Virus Tat Kill Uninfected CD4+ Cells by a Tumor Necrosis Factor-Related Apoptosis-Induced Ligand-Mediated Mechanism. J. Virol..

[37] Herbeuval J.-P., Lambert C., Sabido O., Cottier M.L., Fournel P., Dy M., Genin C. (2003). Macrophages From Cancer Patients: Analysis of TRAIL, TRAIL Receptors, and Colon Tumor Cell Apoptosis. J. Nat. Cancer Inst..

[38] Chaudhary P.M., Eby M., Jasmin A., Bookwalter A., Murray J., Hood L. (1997). Death Receptor 5, a New Member of the TNFR Family, and DR4 Induce FADD-Dependent Apoptosis and Activate the NF-kB Pathway. Immunity.

[39] Wiley S.R., Schooley K., Smolak P.J., Din W.S., Huang C.P., Nicholl J.K., Sutherland R., Smith T.D., Rauch C., Smith C.A. (1995). Identification and characterization of a new member of the TNF family that induces apoptosis. Immunity.

[40] Siegal F.P., Kadowaki N., Shodell M., Fitzgerald-Bocarsly P.A., Shah K., Ho S., Antonenko S., Liu Y.J. (1999). The Nature of the Principal Type 1 Interferon-Producing Cells in Human Blood. Science.

[41] Wilen C.B., Tilton J.C., Doms R.W. (2012). Molecular mechanisms of HIV entry. Adv. Exp. Med. Biol..

[42] Caruz A., Samsom M., Alonso J.M., Alcami J., Baleux F., Virelizier J.L., Parmentier M., Arenzana-Seisdedos F. (1998). Genomic organization and promoter characterization of human CXCR4 gene. FEBS Lett..

[43] Yao Q., Compans R.W., Chen C. (2001). HIV Envelope Proteins Differentially Utilize CXCR4 and CCR5 Coreceptors for Induction of Apoptosis. Virology.

[44] Garg H., Blumenthal R. (2008). Role of HIV Gp41 mediated fusion/hemifusion in bystander apoptosis. Cell. Mol. Life Sci..

[45] Joshi A., Nyakeriga A.M., Ravi R., Garg H. (2011). HIV ENV Glycoprotein-mediated Bystander Apoptosis Depends on Expression of the CCR5 Co-receptor at the Cell Surface and ENV Fusogenic Activity. J. Biol. Chem..

[46] Frankel A.D., Young J.A.T. (1998). HIV-1: Fifteen Proteins and an RNA. Annu. Rev. Biochem..

[47] Naghavi M.H., Goff S.P. (2007). Retroviral proteins that interact with the host cell cytoskeleton. Curr. Opin. Immunol..

[48] Fevrier M., Dorgham K., Rebollo A. (2011). CD4+ T cell depletion in human immunodeficiency virus (HIV) infection: Role of apoptosis. Viruses.

[49] Cossarizza A. (2008). Apoptosis and HIV Infection: About Molecules and Genes. Curr. Pharm. Des..

[50] Honer B., Shoeman R.L., Traub P. (1991). Human immunodeficiency virus type 1 protease microinjected into cultured human skin fibroblasts cleaves vimentin and affects cytoskeletal and nuclear architecture. J. Cell Sci..

[51] Shoeman R.L., Hüttermann C., Hartig R., Traub P. (2001). Amino-terminal Polypeptides of Vimentin Are Responsible for the Changes in Nuclear Architecture Associated with Human Immunodeficiency Virus Type 1 Protease Activity in Tissue Culture Cells. Mol. Biol. Cell..

[52] Blanco R., Carrasco L., Ventoso I. (2003). Cell Killing by HIV-1 Protease. J. Biol. Chem..

[53] Strack P.R., Frey M.W., Rizzo C.J., Cordova B., George H.J., Meade R., Ho S.P., Corman J., Tritch R., Korant B.D. (1996). Apoptosis mediated by HIV protease is preceded by cleavage of Bcl-2. Proc. Natl. Acad. Sci. USA.

[54] Nie Z., Bren G.D., Vlahakis S.R., Schimnich A.A., Brenchley J.M., Trushin S.A., Warren S., Schnepple D.J., Kovacs C.M., Loutfy M.R. (2007). Human Immunodeficiency Virus Type 1 Protease Cleaves Procaspase 8 *In Vivo*. J. Virol..

[55] Riviere Y., Blank V., Kourilsky P., Israel A. (1991). Processing of the precursor of NF-κB by the HIV-1 protease during acute infection. Nature.

[56] Bren G.D., Whitman J., Cummins N., Shepard B., Rizza S.A., Trushin S.A., Badley A.D. (2008). Infected Cell Killing by HIV-1 Protease Promotes NF-κB Dependent HIV-1 Replication. PLoS One.

[57] Bartz S.R., Emerman M. (1999). Human Immunodeficiency Virus Type 1 Tat Induces Apoptosis and Increases Sensitivity to Apoptotic Signals by Up-Regulating FLICE/Caspase-8. J. Virol..

[58] Cummins N.W., Badley A.D. (2010). Mechanisms of HIV-associated lymphocyte apoptosis: 2010. Cell Death Dis..

[59] Dabrowska A., Kim N., Aldovini A. (2008). Tat-Induced FOXO3a Is a Key Mediator of Apoptosis in HIV-1-Infected Human CD4+ T Lymphocytes. J. Immunol..

[60] Skurk C., Maatz H., Kim H.-S., Yang J., Abid M.R., Aird W.C., Walsh K. (2004). The Akt-regulated Forkhead Transcription Factor FOXO3a Controls Endothelial Cell Viability through Modulation of the Caspase-8 Inhibitor FLIP. J. Biol. Chem..

[61] Chen D., Wang M., Zhou S., Zhou Q. (2002). HIV-1 Tat targets microtubules to induce apoptosis, a process promoted by the pro-apoptotic Bcl-2 relative Bim. EMBO J..

[62] Huo L., Li D., Sun L., Liu M., Shi X., Sun X., Li J., Dong B., Dong X., Zhou J. (2011). Tat acetylation regulates its actions on microtubule dynamics and apoptosis in T lymphocytes. J. Pathol..

[63] Buccigrossi V., Laudiero G., Nicastro E., Miele E., Esposito F., Guarino A. (2011). The HIV-1 Transactivator Factor (Tat) Induces Enterocyte Apoptosis through a Redox-Mediated Mechanism. PLoS One.

[64] Gibellini D., Carla Re M., Ponti C., Vitone F., Bon I., Fabbri G., Grazia Di Iasio M., Zauli G. (2005). HIV-1 Tat protein concomitantly down-regulates apical caspase-10 and up-regulates c-FLIP in lymphoid T cells: A potential molecular mechanism to escape TRAIL cytotoxicity. J. Cell. Physiol..

[65] Zheng L., Yang Y., Guocai L., Pauza D., Salvatob M.S. (2007). HIV Tat Protein Increases Bcl-2 Expression in Monocytes Which Inhibits Monocyte Apoptosis Induced by Tumor Necrosis Factor-Alpha-Related Apoptosis-Induced Ligand. Intervirology.

[66] Ekoff M., Kaufmann T., Engström M., Motoyama N., Villunger A., Jönsson J.-I., Strasser A., Nilsson G. (2007). The BH3-only protein Puma plays an essential role in cytokine deprivation-induced apoptosis of mast cells. Blood.

[67] Zauli G., Gibellini D., Secchiero P., Dutartre H.L.N., Olive D., Capitani S., Collette Y. (1999). Human Immunodeficiency Virus Type 1 Nef Protein Sensitizes CD4+ T Lymphoid Cells to Apoptosis via Functional Upregulation of the CD95/CD95 Ligand Pathway. Blood.

[68] Lee S.B., Park J., Jung J.U., Chung J. (2005). Nef induces apoptosis by activating JNK signaling pathway and inhibits NF-κB-dependent immune responses in Drosophila. J. Cell. Sci..

[69] Rasola A., Gramaglia D., Boccaccio C., Comoglio P.M. (2001). Apoptosis Enhancement by the HIV-1 Nef Protein. J. Immunol..

[70] James C.O., Huang M.-B., Khan M., Garcia-Barrio M., Powell M.D., Bond V.C. (2004). Extracellular Nef Protein Targets CD4+ T Cells for Apoptosis by Interacting with CXCR4 Surface Receptors. J. Virol..

[71] Yoon K., Jeong J.G., Kim S. (2001). Stable expression of human immunodeficiency virus type 1 Nef confers resistance against Fas-mediated apoptosis. AIDS Res. Hum. Retrovir..

[72] Geleziunas R., Xu W., Takeda K., Ichijo H., Greene W.C. (2001). HIV-1 Nef inhibits ASK1-dependent death signalling providing a potential mechanism for protecting the infected host cell. Nature.

[73] Wolf D., Witte V., Laffert B., Blume K., Stromer E., Trapp S., d'Aloja P., Schurmann A., Baur A.S. (2001). HIV-1 Nef associated PAK and PI3-Kinases stimulate Akt-independent Bad-phosphorylation to induce anti-apoptotic signals. Nat. Med..

[74] Olivetta E., Federico M. (2006). HIV-1 Nef protects human-monocyte-derived macrophages from HIV-1-induced apoptosis. Exp. Cell. Res..

[75] Robichaud G.A., Poulin L. (2000). HIV type 1 nef gene inhibits tumor necrosis factor alpha-induced apoptosis and promotes cell proliferation through the action of MAPK and JNK in human glial cells. AIDS Res. Hum. Retrovir..

[76] Greenway A.L., McPhee D.A., Allen K., Johnstone R., Holloway G., Mills J., Azad A., Sankovich S., Lambert P. (2002). Human Immunodeficiency Virus Type 1 Nef Binds to Tumor Suppressor p53 and Protects Cells against p53-Mediated Apoptosis. J. Virol..

[77] Xu X.-N., Laffert B., Screaton G.R., Kraft M., Wolf D., Kolanus W., Mongkolsapay J., McMichael A.J., Baur A.S. (1999). Induction of Fas Ligand Expression by HIV Involves the Interaction of Nef with the T Cell Receptor ζ Chain. J. Exp. Med..

[78] van den Broeke C., Radu M., Chernoff J., Favoreel H.W. (2011). An emerging role for p21-activated kinases (Paks) in viral infections. Trends Cell Biol..

[79] Abbas W., Khan K.A., Kumar A., Tripathy M.K., Dichamp I., Keita M., Mahlknecht U., Rohr O., Herbein G. (2014). Blockade of BFA-mediated apoptosis in macrophages by the HIV-1 Nef protein. Cell Death Dis..

[80] Kwon H.-S., Brent M.M., Getachew R., Jayakumar P., Chen L.-F., Schnolzer M., McBurney M.W., Marmorstein R., Greene W.C., Ott M. (2008). Human Immunodeficiency Virus Type 1 Tat Protein Inhibits the SIRT1 Deacetylase and Induces T Cell Hyperactivation. Cell Host Microbe..

[81] Lara H.H., Ixtepan-Turrent L., Garza-Trevino E.N., Badillo-Almaraz J.I., Rodriguez-Padilla C. (2011). Antiviral mode of action of bovine dialyzable leukocyte extract against human immunodeficiency virus type 1 infection. BMC Res. Notes.

[82] Ganau M., Prisco L., Pescador D., Ganau L. (2012). Challenging new targets for CNS-HIV infection. Front. Neurol..

[83] Akari H., Bour S., Kao S., Adachi A., Strebel K. (2001). The Human Immunodeficiency Virus Type 1 Accessory Protein Vpu Induces Apoptosis by Suppressing the Nuclear Factor kB dependent Expression of Antiapoptotic Factors. J. Exp. Med..

[84] Verma S., Ali A., Arora S., Banerjea A.C. (2011). Inhibition of β-TrcP- dependent ubiquitination of p53 by HIV-1 Vpu promotes p53- mediated apoptosis in human T cells. Blood.

[85] Casella C.R., Rapaport E.L., Finkel T.H. (1999). Vpu Increases Susceptibility of Human Immunodeficiency Virus Type 1-Infected Cells to Fas Killing. J. Virol..

[86] Marchal C., Vinatier G., Sanial M., Plessis A., Pret A.M., Limbourg-Bouchon B., Theodore L., Netter S. (2012). The HIV-1 Vpu protein induces apoptosis in Drosophila via activation of JNK signaling. PLoS One.

[87] Komoto S., Tsuji S., Ibrahim M.S., Li Y.-G., Warachit J., Taniguchi K., Ikuta K. (2003). The Vpu Protein of Human Immunodeficiency Virus Type 1 Plays a Protective Role against Virus-Induced Apoptosis in Primary CD4+ T Lymphocytes. J. Virol..

[88] Tateyama M., Oyaizu N., McCloskey T.W., Than S., Pahwa S. (2000). CD4 T lymphocytes are primed to express Fas ligand by CD4 cross-linking and to contribute to CD8 T-cell apoptosis via Fas/FasL death signaling pathway. Blood.

[89] Somma F., Tuosto L., Montani M.S.G., Di Somma M.M., Cundari E., Piccolella E. (2000). Engagement of CD4 Before TCR Triggering Regulates Both Bax- and Fas (CD95)-Mediated Apoptosis. J. Immunol..

[90] Trushin S.A., Algeciras-Schimnich A., Vlahakis S.R., Bren G.D., Warren S., Schnepple D.J., Badley A.D. (2007). Glycoprotein 120 Binding to CXCR4 Causes p38-Dependent Primary T Cell Death That Is Facilitated by, but Does Not Require Cell-Associated CD4. J. Immunol..

[91] Herbein G., Mahlknecht U., Batliwalla F., Gregersen P., Pappas T., Butler J., O'Brien W.A., Verdin E. (1998). Apoptosis of CD8+ T cells is mediated by macrophages through interaction of HIV gp120 with chemokine receptor CXCR4. Nature.

[92] Blanco J., Barretina J., Ferri K.F., Jacotot E., Gutierrez A., Armand-Ugon M., Cabrera C., Kroemer G., Clotet B., Este J. (2003). Cell-Surface-Expressed HIV-1 Envelope Induces the Death of CD4 T Cells during GP41-Mediated Hemifusion-like Events. Virology.

[93] Perfettini J.L., Castedo M., Roumier T., Andreau K., Nardacci R., Piacentini M., Kroemer G. (2005). Mechanisms of apoptosis induction by the HIV-1 envelope. Cell Death Differ..

[94] Garg H., Blumenthal R. (2006). HIV gp41-induced apoptosis is mediated by caspase-3-dependent mitochondrial depolarization, which is inhibited by HIV protease inhibitor nelfinavir. J. Leukoc. Biol..

[95] Castedo M., Roumier T., Blanco J., Ferri K.F., Barretina J., Tintignac L.A., Andreau K., Perfettini J.L., Amendola A., Nardacci R. (2002). Sequential involvement of Cdk1, mTOR and p53 in apoptosis induced by the HIV-1 envelope. Embo. J..

[96] Perfettini J.-L., Roumier T., Castedo M., Larochette N., Boya P., Raynal B., Lazar V., Ciccosanti F., Nardacci R., Penninger J. (2004). HF-КB and p53 Are the Dominant Apoptosis-inducing Transcription Factors Elicited by the HIV-1 Envelope. J. Exp. Med..

[97] Szawlowski P.W.S., Hanke T., Randall R.E. (1993). Sequence homology between HIV-1 gp120 and the apoptosis mediating protein Fas. AIDS.

[98] Silvestris F., Nagata S., Cafforio P., Silvestris N., Dammacco F. (1996). Cross-linking of Fas by antibodies to a peculiar domain of gp120 V3 loop can enhance T cell apoptosis in HIV-1-infected patients. J. Exp. Med..

[99] Michalski C., Li Y., Kang C.Y. (2010). Induction of cytopathic effects and apoptosis in *Spodoptera. frugiperda* cells by the HIV-1 Env glycoprotein signal peptide. Virus Genes..

[100] Koga Y., Sasaki M., Nakamura K., Kimura G., Nomoto K. (1990). Intracellular distribution of the envelope glycoprotein of human immunodeficiency virus and its role in the production of cytopathic effect in CD4+ and CD4- human cell lines. J. Virol..

[101] Andreau K., Perfettini J.-L., Castedo M., Metivier D., Scott V., Pierron G., Kroemer G. (2004). Contagious apoptosis facilitated by the HIV-1 envelope: Fusion-induced cell-to-cell transmission of a lethal signal. J. Cell. Sci..

[102] LaBonte J.A., Madani N., Sodroski J. (2003). Cytolysis by CCR5-Using Human Immunodeficiency Virus Type 1 Envelope Glycoproteins Is Dependent on Membrane Fusion and Can Be Inhibited by High Levels of CD4 Expression. J. Virol..

[103] Lenassi M., Cagney G., Liao M., Vaupotič T., Bartholomeeusen K., Cheng Y., Krogan N.J., Plemenitaš A., Peterlin B.M. (2010). HIV Nef is Secreted in Exosomes and Triggers Apoptosis in Bystander CD4+ T Cells. Traffic.

[104] Fujii Y., Otake K., Tashiro M., Adachi A. (1996). Human immunodeficiency virus type 1 Nef protein on the cell surface is cytocidal for human CD4+ T cells. FEBS Lett..

[105] Fujii Y., Otake K., Tashiro M., Adachi A. (1996). Soluble Nef antigen of HIV-1 is cytotoxic for human CD4+ T cells. FEBS Lett..

[106] Otake K., Fujii Y., Nakaya T., Nishino Y., Zhong Q., Fujinaga K., Kameoka M., Ohki K., Ikuta K. (1994). The carboxyl-terminal region of HIV-1 Nef protein is a cell surface domain that can interact with CD4+ T cells. J. Immunol..

[107] Okada H., Takei R., Tashiro M. (1997). Nef protein of HIV-1 induces apoptotic cytolysis of murine lymphoid cells independently of CD95 (Fas) and its suppression by serine/threonine protein kinase inhibitors. FEBS Lett..

[108] Kumawat K., Pathak S.K., Spetz A.-L., Kundu M., Basu J. (2010). Exogenous Nef Is an Inhibitor of Mycobacterium tuberculosis-induced Tumor Necrosis Factor-α Production and Macrophage Apoptosis. J. Biol. Chem..

[109] Sawai E.T., Baur A., Struble H., Peterlin B.M., Levy J.A., Cheng-Mayer C. (1994). Human immunodeficiency virus type 1 Nef associates with a cellular serine kinase in T lymphocytes. Proc. Natl. Acad Sci. USA.

[110] Renkema G.H., Manninen A., Mann D.A., Harris M., Saksela K. (1999). Identification of the Nef-associated kinase as p21-activated kinase 2. Curr. Biol..

[111] Arora V.K., Fredericksen B.L., Garcia J.V. (2002). Nef: Agent of cell subversion. Microbes Infect..

[112] Mateyak M.K., Kinzy T.G. (2010). eEF1A: Thinking outside the ribosome. J. Biol. Chem..

[113] Debaisieux S., Rayne F., Yezid H., Beaumelle B. (2011). The Ins and Outs of HIV-1 Tat. Traffic.

[114] McCloskey T.W., Ott M., Tribble E., Khan S.A., Teichberg S., Paul M.O., Pahwa S., Verdin E., Chirmule N. (1997). Dual role of HIV Tat in regulation of apoptosis in T cells. J. Immunol..

[115] Badou A., Bennasser Y., Moreau M., Leclerc C., Benkirane M., Bahraoui E. (2000). Tat Protein of Human Immunodeficiency Virus Type 1 Induces Interleukin-10 in Human Peripheral Blood Monocytes: Implication of Protein Kinase C-Dependent Pathway. J. Virol..

[116] Reinhold D., Wrenger S., KÃhne T., Ansorge S. (1999). HIV-1 Tat: Immunosuppression via TGF-β1 induction. Immunol. Today.

[117] Westendorp M.O., Frank R., Ochsenbauer C., Stricker K., Dhein J., Walczak H., Debatin K.M., Krammer P.H. (1995). Sensitization of T cells to CD95-mediated apoptosis by HIV-1 Tat and gp120. Nature.

[118] Minami R., Yamamoto M., Takahama S., Miyamura T., Watanabe H., Suematsu E. (2006). RCAS1 induced by HIV-Tat is involved in the apoptosis of HIV-1 infected and uninfected CD4+ T cells. Cell. Immunol..

[119] Terwilliger E.F., Cohen E.A., Lu Y.C., Sodroski J.G., Haseltine W.A. (1989). Functional role of human immunodeficiency virus type 1 vpu. Proc. Natl. Acad. Sci. USA.

[120] Dube M., Bego M.G., Paquay C., Cohen E.A. (2010). Modulation of HIV-1-host interaction: Role of the Vpu accessory protein. Retrovirology.

[121] Schindler M., Rajan D., Banning C., Wimmer P., Koppensteiner H., Iwanski A., Specht A., Sauter D., Dobner T., Kirchhoff F. (2010). Vpu serine 52 dependent counteraction of tetherin is required for HIV-1 replication in macrophages, but not in *ex vivo ex vivo* human lymphoid tissue. Retrovirology.

[122] Andersen J.L., Le Rouzic E., Planelles V. (2008). HIV-1 Vpr: Mechanisms of G2 arrest and apoptosis. Exp. Mol. Path..

[123] Roshal M., Kim B., Zhu Y., Nghiem P., Planelles V. (2003). Activation of the ATR-mediated DNA Damage Response by the HIV-1 Viral Protein R. J. Biol. Chem..

[124] Yuan H., Xie Y.-M., Chen I.S.Y. (2003). Depletion of Wee-1 Kinase Is Necessary for both Human Immunodeficiency Virus Type 1 Vpr- and Gamma Irradiation-Induced Apoptosis. J. Virol..

[125] DeHart J.L., Zimmerman E.S., Ardon O., Monteiro-Filho C.M., Arganaraz E.R., Planelles V. (2007). HIV-1 Vpr activates the G2 checkpoint through manipulation of the ubiquitin proteasome system. Virol. J..

[126] Snyder A., Alsauskas Z.C., Leventhal J.S., Rosenstiel P.E., Gong P., Chan J.J., Barley K., He J.C., Klotman M.E., Ross M.J. (2010). HIV-1 viral protein r induces ERK and caspase-8-dependent apoptosis in renal tubular epithelial cells. AIDS.

[127] Cagnol S., Van Obberghen-Schilling E., Chambard J.C. (2006). Prolonged activation of ERK1,2 induces FADD-independent caspase 8 activation and cell death. Apoptosis.

[128] Muthumani K., Hwang D.S., Desai B.M., Zhang D., Dayes N., Green D.R., Weiner D.B. (2002). HIV-1 Vpr Induces Apoptosis through Caspase 9 in T Cells and Peripheral Blood Mononuclear Cells. J. Biol. Chem..

[129] Muthumani K., Zhang D., Hwang D.S., Kudchodkar S., Dayes N.S., Desai B.M., Malik A.S., Yang J.S., Chattergoon M.A., Maguire H.C. (2002). Adenovirus encoding HIV-1 Vpr activates caspase 9 and induces apoptotic cell death in both p53 positive and negative human tumor cell lines. Oncogene.

[130] Zhu Y., Gelbard H.A., Roshal M., Pursell S., Jamieson B.D., Planelles V. (2001). Comparison of Cell Cycle Arrest, Transactivation, and Apoptosis Induced by the Simian Immunodeficiency Virus SIVagm and Human Immunodeficiency Virus Type 1 vpr Genes. J. Virol..

[131] Jacotot E., Ferri K.F., El Hamel C., Brenner C., Druillennec S., Hoebeke J., Rustin P., Metivier D., Lenoir C., Geuskens M. (2001). Control of Mitochondrial Membrane Permeabilization by Adenine Nucleotide Translocator Interacting with HIV-1 Viral Protein R and Bcl-2. J. Exp. Med..

[132] Roumier T., Vieira H.L., Castedo M., Ferri K.F., Boya P., Andreau K., Druillennec S., Joza N., Penninger J.M., Roques B. (2002). The C-terminal moiety of HIV-1 Vpr induces cell death via a caspase-independent mitochondrial pathway. Cell Death Differ..

[133] Andersen J.L., DeHart J.L., Zimmerman E.S., Ardon O., Kim B., Jacquot G., Benichou S., Planelles V. (2006). HIV-1 Vpr-Induced Apoptosis Is Cell Cycle Dependent and Requires Bax but Not ANT. PLoS Pathog..

[134] Zhu Y., Roshal M., Li F., Blackett J., Planelles V. (2003). Upregulation of survivin by HIV-1 Vpr. Apoptosis.

[135] Conti L., Matarrese P., Varano B., Gauzzi M.C., Sato A., Malorni W., Belardelli F., Gessani S. (2000). Dual Role of the HIV-1 Vpr Protein in the Modulation of the Apoptotic Response of T Cells. J. Immunol..

[136] Guha D., Nagilla P., Redinger C., Srinivasan A., Schatten G.P., Ayyavoo V. (2012). Neuronal apoptosis by HIV-1 Vpr: Contribution of proinflammatory molecular networks from infected target cells. J. Neuroinflamm..

[137] Kim K., Heo K., Choi J., Jackson S., Kim H., Xiong Y., Ari W. (2012). Vpr-Binding Protein Antagonizes p53-Mediated Transcription via Direct Interaction with H3 Tail. Mol. Cell Biol..

[138] Chirmule N., Pahwa S. (1996). Envelope glycoproteins of human immunodeficiency virus type 1: Profound influences on immune functions. Microbiol. Rev..

[139] Garg H., Joshi A., Ye C., Shankar P., Manjunath N. (2011). Single amino acid change in gp41 region of HIV-1 alters bystander apoptosis and CD4 decline in humanized mice. Virol. J..

[140] Ferri K.F., Jacotot E., Blanco J., Este J.A., Zamzami N., Susin S.A., Xie Z., Brothers G., Reed J.C., Penninger J.M. (2000). Apoptosis Control in Syncytia Induced by the HIV Type-1 Envelope Glycoprotein Complex. J. Exp. Med..

[141] Melki M.T., Saidi H., Dufour A., Olivo-Marin J.C., Gougeon M.L. (2010). Escape of HIV-1-infected dendritic cells from TRAIL-mediated NK cell cytotoxicity during NK-DC cross-talk—A pivotal role of HMGB1. PLoS Pathog..

[142] Donaghy H., Pozniak A., Gazzard B., Qazi N., Gilmour J., Gotch F., Patterson S. (2001). Loss of blood CD11c(+) myeloid and CD11c(–) plasmacytoid dendritic cells in patients with HIV-1 infection correlates with HIV-1 RNA virus load. Blood.

[143] Geijtenbeek T.B.H., Kwon D.S., Torensma R., van Vliet S.J., van Duijnhoven G.C.F., Middel J., Cornelissen I.L.M.H.A., Nottet H.S.L.M., KewalRamani V.N., Littman D.R. (2000). DC-SIGN, a Dendritic Cell-Specific HIV-1-Binding Protein that Enhances trans-Infection of T Cells. Cell.

[144] Favaloro B., Allocati N., Graziano V., di Ilio C., de Laurenzi V. (2012). Role of apoptosis in disease. Aging.

[145] Kumar A., Herbein G. (2014). The macrophage: A therapeutic target in HIV-1 infection. Mol. Cell Ther..

[146] Cummins N.W., Jiang W., McGinty J., Bren G.D., Bosch R.J., Landay A., Deeks S.G., Martin J.N., Douek D., Lederman M.M. (2010). Intracellular Casp8p41 Content Is Inversely Associated with CD4 T Cell Count. J. Infect. Dis..

[147] Natesampillai S., Nie Z., Cummins N.W., Jochmans D., Bren G.D., Angel J.B., Badley A.D. (2010). Patients with Discordant Responses to Antiretroviral Therapy Have Impaired Killing of HIV-Infected T Cells. PLoS Pathog..

[148] Sainski A.M., Natesampillai S., Cummins N.W., Bren G.D., Taylor J., Saenz D.T., Poeschla E.M., Badley A.D. (2011). The HIV-1-Specific Protein Casp8p41 Induces Death of Infected Cells through Bax/Bak. J. Virol..

[149] Stary G., Klein I., Kohlhofer S., Koszik F., Scherzer T., Müllauer L., Quendler H., Kohrgruber N., Stingl G. (2009). Plasmacytoid dendritic cells express TRAIL and induce CD4+ T-cell apoptosis in HIV-1 viremic patients. Blood.

[150] Kawamura T., Gatanaga H., Borris D.L., Connors M., Mitsuya H., Blauvelt A. (2003). Decreased Stimulation of CD4+ T Cell Proliferation and IL-2 Production by Highly Enriched Populations of HIV-Infected Dendritic Cells. J. Immunol..

[151] Doitsh G., Cavrois M., Lassen K.G., Zepeda O., Yang Z., Santiago M.L., Hebbeler A.M., Greene W.C. (2010). Abortive HIV Infection Mediates CD4 T Cell Depletion and Inflammation in Human Lymphoid Tissue. Cell.

[152] Monroe K.M., Yang Z., Johnson J.R., Geng X., Doitsh G., Krogan N.J., Greene W.C. (2014). IFI16 DNA Sensor Is Required for Death of Lymphoid CD4 T Cells Abortively Infected with HIV. Science.

[153] Fink S.L., Cookson B.T. (2005). Apoptosis, Pyroptosis, and Necrosis: Mechanistic Description of Dead and Dying Eukaryotic Cells. Infect. Immun..

[154] Cooper A., Garcia M., Petrovas C., Yamamoto T., Koup R.A., Nabel G.J. (2013). HIV-1 causes CD4 cell death through DNA-dependent protein kinase during viral integration. Nature.

[155] Sakurai Y., Komatsu K., Agematsu K., Matsuoka M. (2009). DNA double strand break repair enzymes function at multiple steps in retroviral infection. Retrovirology.

[156] Verani A., Gras G., Pancino G. (2005). Macrophages and HIV-1: Dangerous liaisons. Mol Immunol..

[157] Wang X., Ye L., Hou W., Zhou Y., Wang Y.-J., Metzger D.S., Ho W.Z. (2009). Cellular microRNA expression correlates with susceptibility of monocytes/macrophages to HIV-1 infection. Blood.

[158] Benaroch P., Billard E., Gaudin R., Schindler M., Jouve M. (2010). HIV-1 assembly in macrophages. Retrovirology.

[159] Groot F., Welsch S., Sattentau Q.J. (2008). Efficient HIV-1 transmission from macrophages to T cells across transient virological synapses. Blood.

[160] Porter K.M., Sutliff R.L. (2012). HIV-1, reactive oxygen species, and vascular complications. Free Radic Biol. Med..

[161] Wyatt C.M., Meliambro K., Klotman P.E. (2012). Recent Progress in HIV-Associated Nephropathy. Ann. Rev. Med..

[162] Winston J.A., Bruggeman L.A., Ross M.D., Jacobson J., Ross L., D’Agati V.D., Klotman P.E., Klotman M.E. (2001). Nephropathy and Establishment of a Renal Reservoir of HIV Type 1 during Primary Infection. N. Eng. J. Med..

[163] Kline E.R., Kleinhenz D.J., Liang B., Dikalov S., Guidot D.M., Hart C.M., Jones D.P., Sutliff R.L. (2008). Vascular oxidative stress and nitric oxide depletion in HIV-1 transgenic rats are reversed by glutathione restoration. Am. J. Physiol..

[164] Zafari A.M., Ushio-Fukai M., Akers M., Yin Q., Shah A., Harrison D.G., Taylor W.R., Griendling K.K. (1998). Role of NADH/NADPH Oxidase-Derived H_2_O_2_ in Angiotensin II-Induced Vascular Hypertrophy. Hypertension.

[165] Shankar S.S., Dube M.P. (2004). Clinical aspects of endothelial dysfunction associated with human immunodeficiency virus infection and antiretroviral agents. Cardiovasc. Toxicol..

[166] Irish B.P., Khan Z.K., Jain P., Nonnemacher M.R., Pirrone V., Rahman S., Rajagopalan N., Suchitra J.B., Mostoller K., Wigdahl B. (2009). Molecular Mechanisms of Neurodegenerative Diseases Induced by Human Retroviruses: A Review. Am. J. Infect. Dis..

[167] Zhang Y., Shi Y., Qiao L., Sun Y., Ding W., Zhang H., Li N., Chen D. (2011). Sigma-1 receptor agonists provide neuroprotection against gp120 via a change in bcl-2 expression in mouse neuronal cultures. Brain Res..

[168] The Lancet Infectious Diseases (2013). The challenge of HIV associated neurocognitive disorder. Lancet Infect. Dis..

[169] Glass J.D., Fedor H., Wesselingh S.L., McArthur J.C. (1995). Immunocytochemical quantitation of human immunodeficiency virus in the brain: Correlations with dementia. Ann. Neurol..

[170] Raivich G., Jones L.L., Kloss C.U.A., Werner A., Neumann H., Kreutzberg G.W. (1998). Immune Surveillance in the Injured Nervous System: T-Lymphocytes Invade the Axotomized Mouse Facial Motor Nucleus and Aggregate around Sites of Neuronal Degeneration. J. Neurosci..

[171] Lipton S.A. (1996). Similarity of neuronal cell injury and death in AIDS dementia and focal cerebral ischemia: Potential treatment with NMDA open-channel blockers and nitric oxide-related species. Brain Pathol..

[172] Sui Z., Fan S., Sniderhan L., Reisinger E., Litzburg A., Schifitto G., Gelbard H.A., Dewhurst S., Maggirwar S.B. (2006). Inhibition of Mixed Lineage Kinase 3 Prevents HIV-1 Tat-Mediated Neurotoxicity and Monocyte Activation. J. Immunol..

[173] Cinque P., Vago L., Mengozzi M., Torri V., Ceresa D., Vicenzi E., Transidico P., Vagani A., Sozzani S., Mantovani A. (1998). Elevated cerebrospinal fluid levels of monocyte chemotactic protein-1 correlate with HIV-1 encephalitis and local viral replication. AIDS.

[174] Nath A. (2002). Human Immunodeficiency Virus (HIV) Proteins in Neuropathogenesis of HIV Dementia. J. Infect. Dis..

[175] Pocernich C.B., Sultana R., Mohmmad-Abdul H., Nath A., Butterfield D.A. (2005). HIV-dementia, Tat-induced oxidative stress, and antioxidant therapeutic considerations. Brain Res. Rev..

[176] Zemlyak I., Sapolsky R., Gozes I. (2009). NAP protects against cytochrome c release: Inhibition of the initiation of apoptosis. Eur. J. Pharmacol..

[177] Jones G.J., Barsby N.L., Cohen Ã.r.A., Holden J., Harris K., Dickie P., Jhamandas J., Power C. (2007). HIV-1 Vpr Causes Neuronal Apoptosis and In Vivo Neurodegeneration. J. Neurosci..

[178] Zink W.E., Zheng J., Persidsky Y., Poluektova L., Gendelman H.E. (1999). The neuropathogenesis of HIV-1 infection. FEMS Immunol. Med. Micro..

[179] Lucas G.M., Eustace J.A., Sozio S., Mentari E.K., Appiah K.A., Moore R.D. (2004). Highly active antiretroviral therapy and the incidence of HIV-1-associated nephropathy: A 12-year cohort study. AIDS.

[180] Zhang G., Liu R., Zhong Y., Plotnikov A.N., Zhang W., Zeng L., Rusinova E., Gerona-Nevarro G., Moshkina N., Joshua J. (2012). Down-regulation of NF-κβ Transcriptional Activity in HIV-associated Kidney Disease by BRD4 Inhibition. J. Biol. Chem..

[181] Bruggeman L.A., Ross M.D., Tanji N., Cara A., Dikman S., Gordon R.E., Burns G.C., D'Agati V.D., Winston J.A., Klotman M.E. (2000). Renal Epithelium Is a Previously Unrecognized Site of HIV-1 Infection. J. Am. Soc. Nephrol..

[182] Mikula M., Fuchs E., Huber H., Beug H., Schulte-Hermann R., Mikulits W. (2004). Immortalized p19ARF null hepatocytes restore liver injury and generate hepatic progenitors after transplantation. Hepatology.

[183] Agati V.D.D., Fogo A.B., Bruijn J.A., Jennette J.C. (2004). Pathologic classification of focal segmental glomerulosclerosis: A working proposal. Am. J. Kidney Dis..

[184] Barisoni L., Kriz W., Mundel P., D’Agati V. (1999). The Dysregulated Podocyte Phenotype. J. Am. Soc. Nephrol..

[185] Bruggeman L.A., Nelson P.J. (2009). Controversies in the pathogenesis of HIV-associated renal diseases. Nat. Rev. Nephrol..

[186] Gharavi A.G., Ahmad T., Wong R.D., Hooshyar R., Vaughn J., Oller S., Frankel R.Z., Bruggeman L.A., D'Agati V.D., Klotman P.E. (2004). Mapping a locus for susceptibility to HIV-1-associated nephropathy to mouse chromosome 3. Proc. Natl. Acad. Sci. USA.

[187] Papeta N., Sterken R., Kiryluk K., Kalyesubula R., Gharavi A. (2011). The molecular pathogenesis of HIV-1 associated nephropathy: Recent advances. J. Mol. Med..

[188] Ross M.J., Martinka S., D’Agati V.D., Bruggeman L.A. (2005). NF-κB Regulates Fas-Mediated Apoptosis in HIV-Associated Nephropathy. J. Am. Soc. Nephrol..

[189] Albaqumi M., Soos T.J., Barisoni L., Nelson P.J. (2006). Collapsing Glomerulopathy. J. Am. Soc. Nephrol..

[190] Zuo Y., Matsusaka T., Zhong J., Ma J., Ma L.-J., Hanna Z., Jolicoeur P., Fogo A.B., Ichikawa I. (2006). HIV-1 Genes vpr and nef Synergistically Damage Podocytes, Leading to Glomerulosclerosis. J. Am. Soc. Nephrol..

[191] Rosenstiel P.E., Gruosso T., Letourneau A.M., Chan J.J., LeBlanc A., Husain M., Najfeld V., Planelles V., D'Agati V.D., Klotman M.E. (2008). HIV-1 Vpr inhibits cytokinesis in human proximal tubule cells. Kidney Int..

[192] Ross M.J., Wosnitzer M.S., Ross M.D., Granelli B., Gusella G.L., Husain M., Kaufman L., Vasievich M., D'Agati V.D., Wilson P.D. (2006). Role of Ubiquitin-Like Protein FAT10 in Epithelial Apoptosis in Renal Disease. J. Am. Soc. Nephrol..

[193] Barbaro G. (2002). Cardiovascular Manifestations of HIV Infection. Circulation.

[194] Krishnaswamy G., Chi D.S., Kelley J.L., Sarubbi F., Smith J.K., Peiris A. (2000). The cardiovascular and metabolic complications of HIV infection. Cardiol. Rev..

[195] Lo J., Plutzky J. (2012). The Biology of Atherosclerosis: General Paradigms and Distinct Pathogenic Mechanisms Among HIV-Infected Patients. J. Infect. Dis..

[196] Diaz P.T., King M.A., Pacht E.R., Wewers M.D., Gadek J.E., Nagaraja H.N., Drake J., Clanton T.L. (2000). Increased Susceptibility to Pulmonary Emphysema among HIV-Seropositive Smokers. Ann. Int. Med..

[197] Crothers K., Butt A.A., Gibert C.L., Rodriguez-Barradas M.C., Crystal S., Justice A.C. (2006). Increased COPD Among HIV-Positive Compared to HIV-Negative Veterans. CHEST J..

[198] Kirk G.D., Merlo C., O’Driscoll P., Mehta S.H., Galai N., Vlahov D., Samet J., Engels E.A. (2007). HIV Infection Is Associated with an Increased Risk for Lung Cancer, Independent of Smoking. Clin. Infect. Dis..

[199] Rusnati M., Presta M. (2002). HIV-1 Tat protein and endothelium: From protein/cell interaction to AIDS-associated pathologies. Angiogenesis.

[200] Kanmogne G.D., Primeaux C., Grammas P. (2005). Induction of apoptosis and endothelin-1 secretion in primary human lung endothelial cells by HIV-1 gp120 proteins. Biochem. Biophys. Res. Comm..

[201] Dezube B.J. (1996). Clinical presentation and natural history of AIDS-related Kaposi’s sarcoma. Hematol. Oncol. Clin. N. Am..

[202] Grayson W., Pantanowitz L. (2008). Histological variants of cutaneous Kaposi sarcoma. Diagn. Pathol..

[203] Suster S., Fisher C., Moran C.A. (1998). Expression of bcl-2 oncoprotein in benign and malignant spindle cell tumors of soft tissue, skin, serosal surfaces, and gastrointestinal tract. Am. J. Surg. Pathol..

[204] Pillay P., Chetty R., Re R. (1999). Bcl-2 and p53 Immunoprofile in Kaposi’s Sarcoma. Pathol. Oncol. Res..

[205] Kaaya E., Castanos-Velez E., Heiden T., Ekman M., Catrina A.I., Kitinya J., Andersson L., Biberfeld P. (2000). Proliferation and apoptosis in the evolution of endemic and acquired immunodeficiency syndrome-related Kaposi’s sarcoma. Med. Oncol..

[206] Mori S., Murakami-Mori K., Nakamura S., Ashkenazi A., Bonavida B. (1999). Sensitization of AIDS-Kaposi’s Sarcoma Cells to Apo-2 Ligand-Induced Apoptosis by Actinomycin D. J. Immunol..

[207] Sgadari C., Barillari G., Palladino C., Bellino S., Taddeo B., Toschi E., Ensoli B. (2011). Fibroblast Growth Factor-2 and the HIV-1 Tat Protein Synergize in Promoting Bcl-2 Expression and Preventing Endothelial Cell Apoptosis: Implications for the Pathogenesis of AIDS-Associated Kaposi’s Sarcoma. Int. J. Vasc. Med..

[208] Nor J.E., Christensen J., Liu J., Peters M., Mooney D.J., Strieter R.M., Polverini P.J. (2001). Up-Regulation of Bcl-2 in Microvascular Endothelial Cells Enhances Intratumoral Angiogenesis and Accelerates Tumor Growth. Cancer Res..

[209] Sakai Y., Goodison S., Kusmartsev S., Fletcher B., Eruslanov E., Cao W., Porvasnik S., Namiki K., Anai S., Rosser C.J. (2009). Bcl-2 mediated modulation of vascularization in prostate cancer xenografts. Prostate.

[210] Michel J.-B. (2003). Anoikis in the Cardiovascular System. Arterioscler. Thromb. Vasc. Biol..

[211] Eccles S.A. (2004). Parallels in invasion and angiogenesis provide pivotal points for therapeutic intervention. Int. J. Dev. Biol..

[212] Almodovar S., Knight R., Allshouse A.A., Roemer S., Lozupone C., McDonald D., Widmann J., Voelkel N.F., Shelton R.J., Suarez E.B. (2012). Human Immunodeficiency Virus Nef signature sequences are associated with pulmonary hypertension. AIDS Res. Hum. Retrovir..

[213] Li C.J., Friedman D.J., Wang C., Metelev V., Pardee A.B. (1995). Induction of apoptosis in uninfected lymphocytes by HIV-1 Tat protein. Science.

[214] Jajoo S., Mukherjea D., Brewer G.J., Ramkumar V. (2008). Pertussis toxin B-oligomer suppresses human immunodeficiency virus-1 Tat-induced neuronal apoptosis through feedback inhibition of phospholipase C-β by protein kinase C. Neuroscience.

[215] Liu H., Yu S., Zhang H., Xu J. (2012). Angiogenesis Impairment in Diabetes: Role of Methylglyoxal-Induced Receptor for Advanced Glycation Endproducts, Autophagy and Vascular Endothelial Growth Factor Receptor 2. PLoS One.

[216] Rizza S.A., Badley A.D. (2008). HIV protease inhibitors impact on apoptosis. Med. Chem..

[217] Vocero-Akbani A.M., Heyden N.V., Lissy N.A., Ratner L., Dowdy S.F. (1999). Killing HIV-infected cells by transduction with an HIV protease-activated caspase-3 protein. Nat. Med..

[218] Miyauchi K., Urano E., Takizawa M., Ichikawa R., Komano J. (2012). Therapeutic potential of HIV protease-activable CASP3. Sci. Rep..

[219] Rehman S., Husain M., Yadav A., Kasinath B.S., Malhotra A., Singhal P.C. (2012). HIV-1 Promotes Renal Tubular Epithelial Cell Protein Synthesis: Role of mTOR Pathway. PLoS One.

[220] Passaes C.P., Sáez-Ciriòn A. (2014). HIV cure research: Advances and prospects. Virology.

[221] Cummins N., Badley A. (2013). Anti-apoptotic mechanisms of HIV: Lessons and novel approaches to curing HIV. Cell. Mol. Life Sci..

[222] Badley A.D., Sainski A., Wightman F., Lewin S.R. (2013). Altering cell death pathways as an approach to cure HIV infection. Cell Death Dis..

[223] Garg H., Joshi A., Blumenthal R. (2009). Altered bystander apoptosis induction and pathogenesis of enfuvirtide-resistant HIV type 1 Env mutants. AIDS Res. Hum. Retrovir..

[224] Chinsembu K.C., Hedimbi M. (2009). A Survey of Plants with Anti-HIV Active Compounds and their Modes of Action. Med. J. Zambia.

[225] Frankel A.D., Bredt D.S., Pabo C.O. (1988). Tat protein from human immunodeficiency virus forms a metal-linked dimer. Science.

[226] Ramautar A., Mabandla M., Blackburn J., Daniels W.M.U. (2012). Inhibition of HIV-1 Tat-induced transactivation and apoptosis by the divalent metal chelators, fusaric acid and picolinic acid Implications for HIV-1 dementia. Neurosci. Res..

[227] De Clercq E. (2009). Anti-HIV drugs: 25 compounds approved within 25 years after the discovery of HIV. Int. J. Antimicrob. Agents.

[228] Judd D.A., Nettles J.H., Nevins N., Snyder J.P., Liotta D.C., Tang J., Ermolieff J., Schinazi R.F., Hill C.L. (2001). Polyoxometalate HIV-1 Protease Inhibitors. A New Mode of Protease Inhibition. J. Am. Chem. Soc..

[229] Sayer J.M., Aniana A., Louis J.M. (2012). Mechanism of Dissociative Inhibition of HIV Protease and Its Autoprocessing from a Precursor. J. Mol. Biol..

[230] Hwang Y.S., Chmielewski J. (2004). A unidirectional crosslinking strategy for HIV-1 protease dimerization inhibitors. Bioorg. Med. Chem. Lett..

[231] Ghosh A.K., Chapsal B.D., Steffey M., Agniswamy J., Wang Y.-F., Amano M., Weber I.T., Mitsuya H. (2012). Substituent effects on P2-cyclopentyltetrahydrofuranyl urethanes: Design, synthesis, and X-ray studies of potent HIV-1 protease inhibitors. Bioorg. Med. Chem. Lett..

[232] Koh Y., Das D., Leschenko S., Nakata H., Ogata-Aoki H., Amano M., Nakayama M., Ghosh A.K., Mitsuya H. (2009). GRL-02031, a novel nonpeptidic protease inhibitor (PI) containing a stereochemically defined fused cyclopentanyltetrahydrofuran potent against multi-PI-resistant human immunodeficiency virus type 1 *in vitro*. Antimicrob. Agents Chemother..

[233] He Y., Xiao Y., Song H., Liang Q., Ju D., Chen X., Lu H., Jing W., Jiang S., Zhang L. (2008). Design and Evaluation of Sifuvirtide, a Novel HIV-1 Fusion Inhibitor. J. Biol. Chem..

[234] Janda E., Visalli V., Colica C., Aprigliano S., Musolino V., Vadalà N., Muscoli C., Sacco I., Iannone M., Rotiroti D. (2011). The protective effect of tianeptine on Gp120-induced apoptosis in astroglial cells: Role of GS and NOS, and NF-κB suppression. Br. J. Pharmacol..

[235] Rider T.H., Zook C.E., Boettcher T.L., Wick S.T., Pancoast J.S., Zusman B.D. (2011). Broad-spectrum antiviral therapeutics. PLoS One.

[236] Thakur B.K., Chandra A., Dittrich T., Welte K., Chandra P. (2012). Inhibition of SIRT1 by HIV-1 viral protein Tat results in activation of p53 pathway. Biochem. Biophys. Res. Comm..

[237] Luo J., Nikolaev A.Y., Imai S.-i., Chen D., Su F., Shiloh A., Guarente L., Gu W. (2001). Negative Control of p53 by SirT1 Promotes Cell Survival under Stress. Cell.

[238] Van Leeuwen I., Lain S., Vande Woude G.F., Klein G. (2009). Sirtuins and p53. Advances in Cancer Research.

[239] Vaziri H., Dessain S.K., Eaton E.N., Imai S.-I., Frye R.A., Pandita T.K., Guarente L., Weinberg R.A. (2001). hSIR2SIRT1 Functions as an NAD-Dependent p53 Deacetylase. Cell.

[240] Peck B., Chen C.Y., Ho K.K., Di Fruscia P., Myatt S.S., Coombes R.C., Fuchter M.J., Hsiao C.D., Lam E.W. (2010). SIRT inhibitors induce cell death and p53 acetylation through targeting both SIRT1 and SIRT2. Mol. Cancer Ther..

[241] Li L., Wang L., Li L., Wang Z., Ho Y., McDonald T., Holyoake T.L., Chen W.Y., Bhatia R. (2012). Activation of p53 by SIRT1 Inhibition Enhances Elimination of CML Leukemia Stem Cells in Combination with Imatinib. Cancer Cell..

[242] Audrito V., Vaisitti T., Rossi D., Gottardi D., D’Arena G., Laurenti L., Gaidano G., Malavasi F., Deaglio S. (2011). Nicotinamide Blocks Proliferation and Induces Apoptosis of Chronic Lymphocytic Leukemia Cells through Activation of the p53/miR-34a/SIRT1 Tumor Suppressor Network. Cancer Res..

[243] Phenix B.N., Angel J.B., Mandy F., Kravcik S., Parato K., Chambers K.A., Gallicano K., Hawley-Foss N., Cassol S., Cameron D.W. (2000). Decreased HIV-associated T cell apoptosis by HIV protease inhibitors. AIDS Res. Hum. Retrovir..

[244] Pope M.T., Müller A. (1994). Polyoxometalates: From Platonic Solids to Anti-Retroviral Activity (Topics in Molecular Organization and Engineering).

[245] Moskovitz B.L. (1988). Clinical trial of tolerance of HPA-23 in patients with acquired immune deficiency syndrome. Antimicrob. Agents Chemo..

[246] Flutsch A., Schroeder T., Grütter M.G., Patzke G.R. (2011). HIV-1 protease inhibition potential of functionalized polyoxometalates. Bioorg. Med. Chem. Lett..

[247] Inouye Y., Tokutake Y., Kunihara J., Yoshida T., Yamase T., Nakata A., Nakamura S. (1992). Suppressive effect of polyoxometalates on the cytopathogenicity of human immunodeficiency virus type 1 (HIV-1) *in vitro* and their inhibitory activity against HIV-1 reverse transcriptase. Chem. Pharm. Bull. (Tokyo).

[248] Gustchina A., Weber I.T. (1991). Comparative analysis of the sequences and structures of HIV-1 and HIV-2 proteases. Proteins.

[249] Tang C., Louis J.M., Aniana A., Suh J.-Y., Clore G.M. (2008). Visualizing transient events in amino-terminal autoprocessing of HIV-1 protease. Nature.

[250] Babé L.M., Rosé J., Craik C.S. (1992). Synthetic “interface” peptides alter dimeric assembly of the HIV 1 and 2 proteases. Protein Sci..

[251] Todd M.J., Semo N., Freire E. (1998). The structural stability of the HIV-1 protease. J. Mol. Biol..

[252] Louis J.M., Ishima R., Torchia D.A., Weber I.T. (2007). HIV-1 Protease: Structure, Dynamics, and Inhibition. Adv. Pharmacol..

[253] Davis D.A., Brown C.A., Singer K.E., Wang V., Kaufman J., Stahl S.J., Wingfield P., Maeda K., Harada S., Yoshimura K. (2006). Inhibition of HIV-1 replication by a peptide dimerization inhibitor of HIV-1 protease. Antivir. Res..

[254] Wei Y., Ma C.-M., Hattori M. (2009). Synthesis and evaluation of A-seco type triterpenoids for anti-HIV-1protease activity. Eur. J. Med. Chem..

[255] Ghosh A.K., Chapsal B.D., Baldridge A., Ide K., Koh Y., Mitsuya H. (2008). Design and Synthesis of Stereochemically Defined Novel Spirocyclic P2-Ligands for HIV-1 Protease Inhibitors. Org. Lett..

[256] Ghosh A.K., Leshchenko-Yashchuk S., Anderson D.D., Baldridge A., Noetzel M., Miller H.B., Tie Y., Wang Y.-F., Koh Y., Weber I.T. (2009). Design of HIV-1 Protease Inhibitors with Pyrrolidinones and Oxazolidinones as Novel P1 Ligands To Enhance Backbone-Binding Interactions with Protease: Synthesis, Biological Evaluation, and Protein Ligand X-ray Studies. J. Med. Chem..

[257] Ghosh A.K., Takayama J. (2008). Enantioselective Synthesis of Cyclopentyltetrahydrofuran (Cp-THF), an Important High-Affinity P2-Ligand for HIV-1 Protease Inhibitors. Tetrahedron Lett..

[258] Mosing R.K., Mendonsa S.D., Bowser M.T. (2005). Capillary Electrophoresis-SELEX Selection of Aptamers with Affinity for HIV-1 Reverse Transcriptase. Anal. Chem..

[259] Baron M., Kinsley N., Sewell B., Jaffer M., Capovilla A., James W., Khati M. Molecular Interaction of gp120 and B40 Aptamer: A Potential New HIV-1 Entry Inhibitor Drug. Proceedings of the 2nd CSIR Biennial Conference: CSIR International Convention Centre Pretoria.

[260] Berges B.K., Akkina S.R., Remling L., Akkina R. (2010). Humanized Rag2−/−γc−/− (RAG-hu) mice can sustain long-term chronic HIV-1 infection lasting more than a year. Virology.

[261] Dey A.K., Griffiths C., Lea S.M., James W. (2005). Structural characterization of an anti-gp120 RNA aptamer that neutralizes R5 strains of HIV-1. RNA.

[262] Liang Y., Zhang Z., Wei H., Hu Q., Deng J., Guo D., Cui Z., Zhang X.-E. (2011). Aptamer beacons for visualization of endogenous protein HIV-1 reverse transcriptase in living cells. Biosens. Bioelectron..

[263] Neff C.P., Zhou J., Remling L., Kuruvilla J., Zhang J., Li H., Smith D.D., Swiderski P., Rossi J.J., Akkina R. (2011). An aptamer-siRNA chimera suppresses HIV-1 viral loads and protects from helper CD4(+) T cell decline in humanized mice. Sci. Transl. Med..

[264] Rahim Ruslinda A., Tanabe K., Ibori S., Wang X., Kawarada H. (2013). Effects of diamond-FET-based RNA aptamer sensing for detection of real sample of HIV-1 Tat protein. Biosens. Bioelectron..

[265] Tombelli S., Minunni M., Luzi E., Mascini M. (2005). Aptamer-based biosensors for the detection of HIV-1 Tat protein. Bioelectrochemistry.

[266] Wheeler L.A., Trifonova R., Vrbanac V., Basar E., McKernan S., Xu Z., Seung E., Deruaz M., Dudek T., Einarsson J.I. (2011). Inhibition of HIV transmission in human cervicovaginal explants and humanized mice using CD4aptamer-siRNA chimeras. J. Clin. Invest..

[267] Zhou J., Shu Y., Guo P., Smith D.D., Rossi J.J. (2011). Dual functional RNA nanoparticles containing phi29 motor pRNA and anti-gp120 aptamer for cell-type specific delivery and HIV-1 Inhibition. Methods.

[268] Zhu Q., Shibata T., Kabashima T., Kai M. (2012). Inhibition of HIV-1 protease expression in T cells owing to DNA aptamer-mediated specific delivery of siRNA. Eur. J. Med. Chem..

[269] Castaldello A., Brocca-Cofano E., Voltan R., Triulzi C., Altavilla G., Laus M., Sparnacci K., Ballestri M., Tondelli L., Fortini C. (2006). DNA prime and protein boost immunization with innovative polymeric cationic core-shell nanoparticles elicits broad immune responses and strongly enhance cellular responses of HIV-1 Tat DNA vaccination. Vaccine.

[270] Chattopadhyay N., Zastre J., Wong H.L., Wu X.Y., Bendayan R. (2008). Solid lipid nanoparticles enhance the delivery of the HIV protease inhibitor, atazanavir, by a human brain endothelial cell line. Pharm. Res..

[271] Weyermann J., Lochmann D., Zimmer A. (2004). Comparison of antisense oligonucleotide drug delivery systems. J. Controll. Release.

[272] Stanberry L.R., Strugnell R. (2011). Vaccines of the future. Perspect. Vaccinol..

[273] Christie R.J., Grainger D.W. (2003). Design strategies to improve soluble macromolecular delivery constructs. Adv. Drug Del. Rev..

[274] Kratz F. (2008). Albumin as a drug carrier: Design of prodrugs, drug conjugates and nanoparticles. J. Contr. Release.

[275] das Neves J., Amiji M.M., Bahia M.F., Sarmento B. (2010). Nanotechnology-based systems for the treatment and prevention of HIV/AIDS. Adv. Drug Del. Rev..

[276] Panyam J., Labhasetwar V. (2003). Biodegradable nanoparticles for drug and gene delivery to cells and tissue. Adv. Drug Del. Rev..

[277] Lobenberg R., Kreuter J. (1996). Macrophage targeting of azidothymidine: A promising strategy for AIDS therapy. AIDS Res. Hum. Retrovir..

[278] Kim S., Scheerer S., Geyer M.A., Howell S.B. (1990). Direct Cerebrospinal Fluid Delivery of an Antiretroviral Agent Using Multivesicular Liposomes. J. Infect. Dis..

